# Understanding the Crystallization of Random Copolymers beyond Iso(di)morphism and Comonomer Exclusion

**DOI:** 10.1021/acs.macromol.6c01061

**Published:** 2026-07-09

**Authors:** Kaojin Wang, Hewei Ding, Ran Chen, Meng Zhang, Yong-Guang Jia, Yaming Wang, Yunxiang Shi, Ricardo A. Pérez-Camargo, Xiao Xia Zhu, Alejandro J. Müller

**Affiliations:** † Department of Chemistry, Faculty of Arts and Sciences, 47836 Beijing Normal University, Zhuhai, Guangdong 519085, China; ‡ Key Laboratory of Materials Processing & Mold, Ministry of Education, National Engineering Research Center for Advanced Polymer Processing Technology, Zhengzhou University, Zhengzhou 450002, China; § POLYMAT and Department of Polymers and Advanced Materials: Physics, Chemistry and Technology, University of the Basque Country UPV/EHU, Paseo Manuel Lardizabal 3, 20018 Donostia-San Sebastián, Spain; ∥ Ikerbasque, Basque Foundation for Science, Plaza Euskadi 5, 48009 Bilbao, Spain

## Abstract

In this Perspective, we revisit the classical classification of crystallization behavior in random copolymers, namely isomorphic crystallization, isodimorphism, and comonomer exclusion, and discuss the limitations of these discrete categories. Although these frameworks describe distinct modes of comonomer inclusion or exclusion within crystalline lattices, growing experimental evidence shows that many copolymer systems exhibit composition- and structure-dependent mixed crystallization behaviors that cannot be fully captured by the traditional classifications. We propose that crystallization in random copolymers should instead be understood as a continuum of structural states. Within this continuum, isomorphism, isodimorphism, comonomer exclusion, and mixed crystallization modes represent distinct pathways by which comonomers are accommodated in crystalline lattices, each yielding distinct structural organizations and material properties. This broader perspective reframes crystallization from a passive consequence of molecular structure into an active materials design tool. By tailoring molecular architecture, copolymer composition, and processing conditions, crystallization behavior can be deliberately manipulated to tune thermal, mechanical, optical, barrier, and degradation properties. Advancing toward predictive control of crystallization in random copolymers will require systematic studies that integrate crystallization mechanisms, advanced structural characterization, and functional performance across diverse polymer systems. Such efforts will enable a transition from empirical classification toward a true crystallization-by-design approach, in which targeted crystalline states and morphologies are engineered to achieve tailored material properties and functionalities.

## Graphical abstract

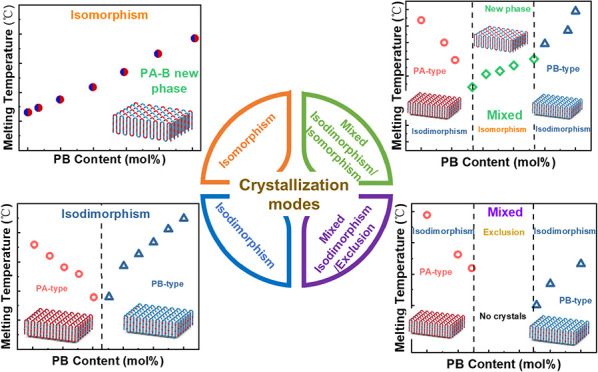

## 1 Introduction

Crystallization, governed by the underlying polymer structure, plays a key role in determining the final properties of semicrystalline polymers, affecting mechanical performance, thermal stability, barrier properties, and degradation behavior. Due to this strong structure-property connection, understanding and controlling polymer crystallization remain important goals in polymer science and materials design. While homopolymers often exhibit relatively clear crystallization behavior, the situation becomes much more complex in random copolymers, where chemically different repeat units introduce structural heterogeneity along the polymer chain.

Random copolymerization is widely used as a molecular design strategy to tailor polymer properties. In contrast to polymer blends, where different components may undergo macrophase separation due to limited thermodynamic miscibility, copolymerization introduces covalent bonds between distinct monomer units along the same polymer chain. This covalent connectivity generally ensures miscibility at the molecular level in both the melt and glassy states. However, crystallization imposes strict structural constraints on the polymer chains because the formation of ordered crystal lattices requires specific conformational and packing arrangements. As a result, the ability of different comonomer units to be incorporated into the crystalline phase is limited, leading to distinct crystallization behaviors.

The crystallization behavior of random copolymers is generally understood to occur in three main modes: *isomorphism, isodimorphism, and comonomer exclusion*.^1–4^ These classical modes have traditionally been treated as distinct crystallization categories, each with characteristic thermal and structural signatures. In isomorphic systems (Figure 1a), both comonomer units can be incorporated into a single crystalline lattice, enabling crystallization across the full range of compositions. In isodimorphic copolymers (Figure 1b), two primary crystal structures are usually observed, each associated with one of the parent homopolymer lattices. Depending on composition, one crystalline phase becomes dominant, while the second comonomer may be partially incorporated as defects within the prevailing crystal structure. At the opposite end, total comonomer exclusion (Figure 1c) happens when the presence of the second unit prevents crystallization over a range of intermediate compositions, resulting in amorphous copolymers within that composition range.

**Figure 1:**
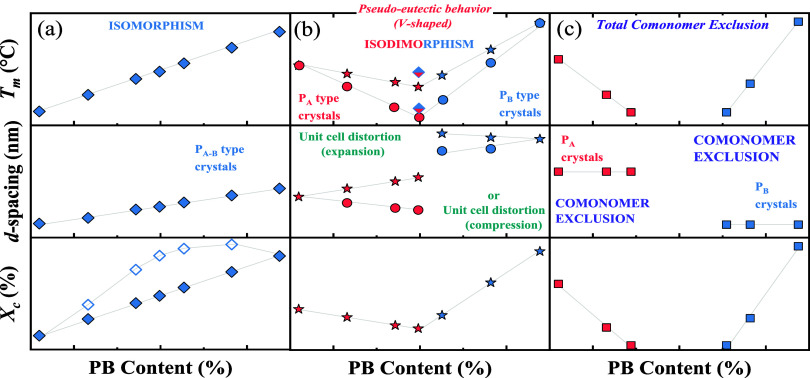
Main crystallization modes in random copolymers: (a) Isomorphism, (b) isodimorphism, and (c) total comonomer exclusion, and their characteristic changes in melting temperature (*T*_m_) (top panel), interplanar distances (*d*-spacing) (middle panel), and degree of crystallinity (*X*_c_) (bottom panel) as a function of PB content for a model PA_*x*_B_*y*_ copolymer. In (a) (bottom panel), open symbols indicate possible deviations from ideal linearity due to strong intermolecular interactions. In isodimorphic systems, pseudoeutectic behavior reflects the balance between comonomer inclusion and exclusion within PA- and PB-like crystals. In (b) (top panel), star and circle symbols represent different degrees of comonomer inclusion within the parent-like lattices. The diamond symbol at the pseudoeutectic composition indicates the possible coexistence of PA- and PB-like crystals, which may lead to two melting transitions. In (b) (middle panel), the star and circle symbols indicate different trends in *d*-spacing evolution associated with unit-cell distortion caused by partial comonomer incorporation. Figure 1 is adapted with permission from ref 19. Copyright 2026 American Chemical Society.

This classical classification has provided a useful framework for understanding the crystallization behavior of many random copolymer systems.^1–4^ However, mounting experimental evidence now shows that this scheme does not fully capture the diversity of crystallization phenomena observed in random copolymers. Some systems combine features of different modes and therefore exhibit mixed crystallization modes^4–10^ (Section 3). These observations suggest transitional crystallization behavior between the classical limits. In parallel, other systems have been classified as isomorphic primarily on the basis of thermal trends, without sufficient structural evidence to demonstrate the existence of a genuinely shared lattice (Section 5).

The central argument of this Perspective is that crystallization in random copolymers should not be interpreted as a collection of isolated categories. Instead, it should be viewed as a continuum of interconnected crystallization states. Within this framework, crystallization behavior is governed by the balance among comonomer inclusion, intermolecular interactions, conformational constraints, and polymorphic accessibility. From this perspective, mixed crystallization modes should not be regarded as anomalous exceptions, but rather as experimentally observable manifestations of transitions between the classical crystallization limits.

Viewing crystallization as a continuum does not invalidate the classical categories of isomorphism, isodimorphism, or comonomer exclusion. Rather, these categories should be understood as dominant or limiting crystallization regimes within a broader structural landscape, linked by transitional and mixed-mode behaviors. This perspective further suggests that the interpretation and classification of crystallization behavior may progressively evolve toward experimentally grounded, ultimately predictive structure-crystallization-property relationships.

In this perspective, we revisit the concepts of cocrystallization, isomorphism, isodimorphism, and comonomer exclusion, focusing on the structural criteria necessary for accurate identification. We also compare representative systems across various polymer families (Section 6) to identify the molecular factors that influence crystallization behavior. Additionally, we discuss the emergence of mixed crystallization modes as signs of transitions between classical modes. Rather than presenting isolated examples organized by polymer family, we use representative systems to construct a comparative framework linking molecular architecture with crystallization behavior. By integrating structural evidence with comparative analysis, this Perspective aims to provide a clearer conceptual basis for interpreting and eventually predicting crystallization behavior in random copolymers.

## 2 Terminology and Structural Criteria: Cocrystallization, Isomorphism, Isodimorphism, and Comonomer Exclusion

Crystallization phenomena in multicomponent polymer systems are often described using terms like cocrystallization, isomorphism, isodimorphism, and comonomer exclusion. Although these concepts are commonly used in the literature, their definitions are not always consistently applied, especially in the context of random copolymers. Clarifying the structural meaning of these terms is therefore essential before discussing the crystallization modes observed in random copolymers.

The concept of cocrystallization generally refers to the formation of a crystalline phase containing more than one molecular species within a shared crystal lattice. In polymer systems, a typical example is the stereocomplex formed in poly­(l-lactic acid)/poly­(d-lactic acid) blends, where two different stereoregular chains assemble into a crystalline structure that is distinct from that of either homopolymer.^11^ In such cases, both molecular species contribute to forming a common crystal lattice, leading to a new crystalline phase with a higher melting point (*T*_m_) than the original homocrystals of PLLA or PDLA.

The term cocrystallization is also widely used in the context of polyolefins, where comonomer units may be incorporated into polyethylene (PE)-like crystal lattices. In these systems, the repeat units are largely composed of −CH_2_– segments, and the structural differences between comonomers are relatively small. As a consequence, incorporation of comonomer units within the crystal lattice is often described as cocrystallization^12–18^ even when inclusion is only partial. In these chemically simple lattices, the concept of well-defined crystallographic defects becomes less straightforward than in more structurally complex polymer systems. In contrast, for many polar polymers such as polyesters, polycarbonates, or polyamides, the presence of chemically distinct functional groups leads to more pronounced structural differences between repeat units. In such systems, the distinction between full incorporation within a common lattice and partial inclusion as defects becomes more clearly defined. For this reason, in the context of random copolymers considered in this Perspective, the term cocrystallization will be used to describe situations in which different comonomer units participate in forming a new common crystalline lattice.

A specific example of cocrystallization in random copolymers is isomorphic crystallization (Figure 1a). In isomorphic copolymers composed of repeat units A and B (PA_*x*_B_*y*_), with *x* and *y* representing their respective compositions, the homopolymers PA and PB crystallize in their own unique crystal structures. However, the copolymer chains crystallize into a common crystalline phase described as a PA_*x*_B_*y*_ lattice. Importantly, this lattice is structurally distinct from the crystal structures of either parent homopolymer (PA and PB) and from any of their polymorphic forms. As a result, crystallization can happen across the entire composition range. Experimentally, this behavior is typically characterized by a continuous, often nearly linear, change in the *T*_m_ with comonomer composition, along with gradual changes in lattice parameters (interplanar distances, *d*-spacing) and degree of crystallinity (*X*_c_). These trends resemble simple rule-of-mixtures behavior, indicating that both comonomer units contribute to the formation of a common crystalline phase.^1–4^ However, a simple rule of mixtures alone is not enough proof of isomorphic behavior. Convincing evidence of isomorphism requires structural proof that the lattice itself evolves gradually rather than merely switching between closely related parent-like phases.

In isodimorphic copolymers (Figure 1b), crystallization occurs in crystal structures similar to those of the parent homopolymers. In this case, the copolymer can crystallize either in PA-like or PB-like crystal lattices, depending on composition and crystallization conditions. The less abundant comonomer unit, which does not determine the formation of the crystallizing lattice, may be incorporated only partially into that structure, causing a slight distortion of the parent lattice. This distortion is reflected in changes in *d*-spacing and in the formation of PA- or PB-like crystals depending on the composition. Consequently, two competing crystalline phases may be observed across the composition range, producing the characteristic pseudoeutectic dependence of *T*_m_ on composition. As the relative stability of the PA-like and PB-like crystalline phases varies with composition, a minimum in *T*_m_ is typically observed near the composition where competition between the two parent-like lattices is maximal. In this pseudoeutectic region, coexistence of both crystalline phases may occur depending on composition, crystallization pathway, and thermal history, resulting in two melting transitions associated with the corresponding parent-like crystals. Concomitantly, the crystallization temperature (*T*_c_) and *X*_c_ also display pseudoeutectic-like trends with composition.

At the opposite extreme is comonomer exclusion (Figure 1c), which happens when the structural mismatch between repeat units stops the minority comonomer from being incorporated into the crystalline lattice of the more abundant one. As a result, only crystals that are essentially identical to those of the parent homopolymers can form when there is enough content of one of the comonomeric units. In this case, crystallization only occurs when the copolymer composition is close to that of the parent homopolymers. As the amount of excluded comonomer increases, crystallization becomes progressively more difficult due to reduced crystallizable sequence length and increased structural perturbation, eventually leading to strongly suppressed or even absent crystallization across intermediate composition ranges. Experimentally, this behavior shows up as the disappearance of crystallization and melting transitions within that composition range. When crystallization still occurs near the homopolymer-rich compositions, the crystal structure remains the same as that of the related homopolymer (PA and PB crystals) because the comonomer units are excluded from the crystalline phase and segregated into the amorphous regions, i.e., they are entirely excluded from the crystal lattice.

The characteristic trends described above represent the behaviors most commonly reported in the literature.^1–4^ However, the amount of available experimental information varies significantly across different crystallization modalities. In particular, isomorphic crystallization seems to be less frequently documented, and several systems described as isomorphic have not been examined with the same level of detailed structural characterization as classical isodimorphic copolymers. Conversely, systems exhibiting comonomer exclusion often receive less attention because the lack of crystallization over broad composition ranges has traditionally been viewed as less advantageous from a materials design perspective. As a result, many of the comparative trends reported in the literature are primarily based on thermal analysis, especially melting and crystallization behaviors obtained through differential scanning calorimetry (DSC), as summarized schematically in Figure 1. However, detailed structural and morphological analyses tend to be less common across all crystallization modes.

In some copolymer systems, crystallization behavior does not fit neatly into any of the three traditional categories. Instead, the dominant crystallization mechanism can change depending on copolymer composition or crystallization conditions, resulting in behaviors that combine features of different crystallization modes. In such cases, transitions between neighboring modes may occur across the composition range, and intermediate regions might display characteristics associated with more than one crystallization modality. These behaviors are commonly called mixed crystallization modes^4–10^ and will be discussed in more detail in Section 3 of this Perspective.

This terminology also suggests a practical hierarchy of evidence. Thermal data provide the first level of classification; structural data determine whether that classification is mechanistically meaningful. Sequence-sensitive methods such as self-nucleation (SN) and successive self-nucleation and annealing (SSA) provide a third level by revealing how inclusion or exclusion affects melt memory and the distributions of crystallizable sequences. This three-level framework is used throughout the rest of this Perspective.

## 3 Mixed Crystallization Modes in Random Copolymers

Recent investigations have shown that the crystallization behavior of random copolymers can be much more complex than the traditional classification of isomorphism, isodimorphism, and total comonomer exclusion suggests. Detailed studies across broad composition ranges have demonstrated that some copolymer families cannot be described by a single crystallization mechanism throughout the full composition range. Instead, different compositional intervals may follow different crystallization pathways, leading to mixed crystallization modes.^4–10^

Importantly, mixed crystallization modes were identified at a stage when semicrystalline copolymers were already under investigation using a broader experimental toolbox. As a result, these systems have often been analyzed using complementary thermal, structural, and sequence-sensitive techniques, including in situ real-time WAXS/SAXS, SN, and SSA, rather than relying exclusively on DSC measurements. These combined approaches revealed that distinct crystallization mechanisms may operate across different compositional regions within the same copolymer family. Consequently, reliable assignment of crystallization modes often requires convergent evidence from thermal, structural, and sequence-sensitive techniques.

A common feature observed in these systems is that, when considering only thermal transitions, the copolymers often show an apparent pseudoeutectic dependence of *T*_m_ (and *T*_c_) on composition (Scheme 1), closely resembling the classical behavior expected for isodimorphic copolymers.^4–10^ As a result, the crystallization behavior might initially be interpreted as traditional isodimorphism based solely on DSC measurements.

**Scheme 1:**
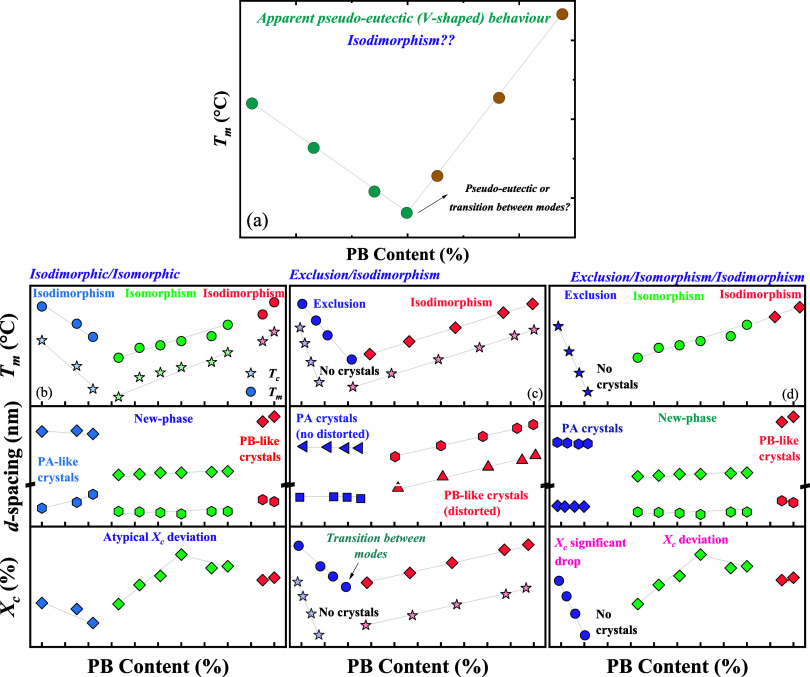
Schematic Comparison between (a) a Classical Pseudo-Eutectic Response and (b–d) Mixed-Mode Cases Leading to Similar Thermal Signatures, Including (b) Isodimorphism/Isomorphism, (c) Isodimorphism/Exclusion, and (d) Triple-Mode^4–10^ Behavior^a^

a Three panels (top-*T*_m_, middle-*d*-spacing, and bottom-*X*_c_) are represented as a function of PB content. In (c), two situations are represented: transition from one region of exclusion to isodimorphism and a similar transition but with a small compositional region of no crystals. The changes in the selected parameters correspond to the reports in the literature.^4–10^ Scheme 1 highlights that fundamentally different crystallization mechanisms can yield very similar pseudo-eutectic thermal signatures, underscoring the limitations of DSC-based classification alone.

However, more detailed analyses reveal that other properties do not necessarily follow the pseudoeutectic trends expected for classical isodimorphism. Deviations may appear in the evolution of *X*_c_ and melting enthalpy (Δ*H*_m_), with values that deviate unexpectedly from the expected V-shaped behavior (Scheme 1b,1d). Structural parameters may also remain nearly unchanged over specific composition ranges, as expected for comonomer exclusion (Scheme 1c,1d). These observations indicate that the underlying crystallization mechanism may vary across the composition range, even when the thermal behavior appears pseudoeutectic. Consequently, structural characterization becomes essential for identifying the actual crystallization mode.^5–8^ Scheme 1 illustrates how the *T*_m_, *d*-spacing, and *X*_c_ vary with PB content for the mixed modes.

The most comprehensive evidence for mixed crystallization modes has so far been reported in random aliphatic copolycarbonates containing octamethylene carbonate (PC8) units, including poly (hexamethylene carbonate-*ran*-PC8) (C6_*x*_C8_*y*_),^5,6,8^ poly (heptamethylene carbonate-*ran*-PC8) (C7_*x*_C8_*y*_),^6,8^ and poly (dodecamethylene carbonate-*ran*-PC8) (C12_*x*_C8_*y*_)^6,8^ copolymers. In all these systems, DSC measurements show a pseudoeutectic dependence of *T*_m_ (and *T*_c_) on composition, seemingly aligning with classical isodimorphism. However, detailed structural analysis using WAXS revealed the formation of an additional crystalline phase (*γ* phase) within specific composition ranges, where unexpectedly high values of Δ*H*_m_ and *X*_c_ were also observed. Importantly, the γ phase could not be assigned to either distorted parent-like crystals or to any known polymorph of the corresponding homopolymers. Instead, the γ phase evolved systematically with copolymer composition, including a continuous variation in *d*-spacing. These observations provided structural evidence for the formation of a genuinely new common lattice involving participation by both comonomer units. This experimental evidence is summarized in Scheme 1b.

In the first reported case, the C6_*x*_C8_*y*_ copolymer (Figure 2) family^5,6,8^ exhibits a γ phase that appears over a wide compositional range (Figure 2c). Within this range, both the *T*_m_ (Figure 2e) and the *d*-spacing (Figure 2d) of the γ phase change consistently with copolymer composition. These results therefore provide clear structural evidence of localized isomorphic crystallization within an otherwise isodimorphic framework. Figure 2 shows the main experimental results, including melt memory (Figure 2g,2h) regarding mixed isodimorphic/isomorphic behavior, using the C6_*x*_C8_*y*_ copolymer as a typical example.

**Figure 2:**
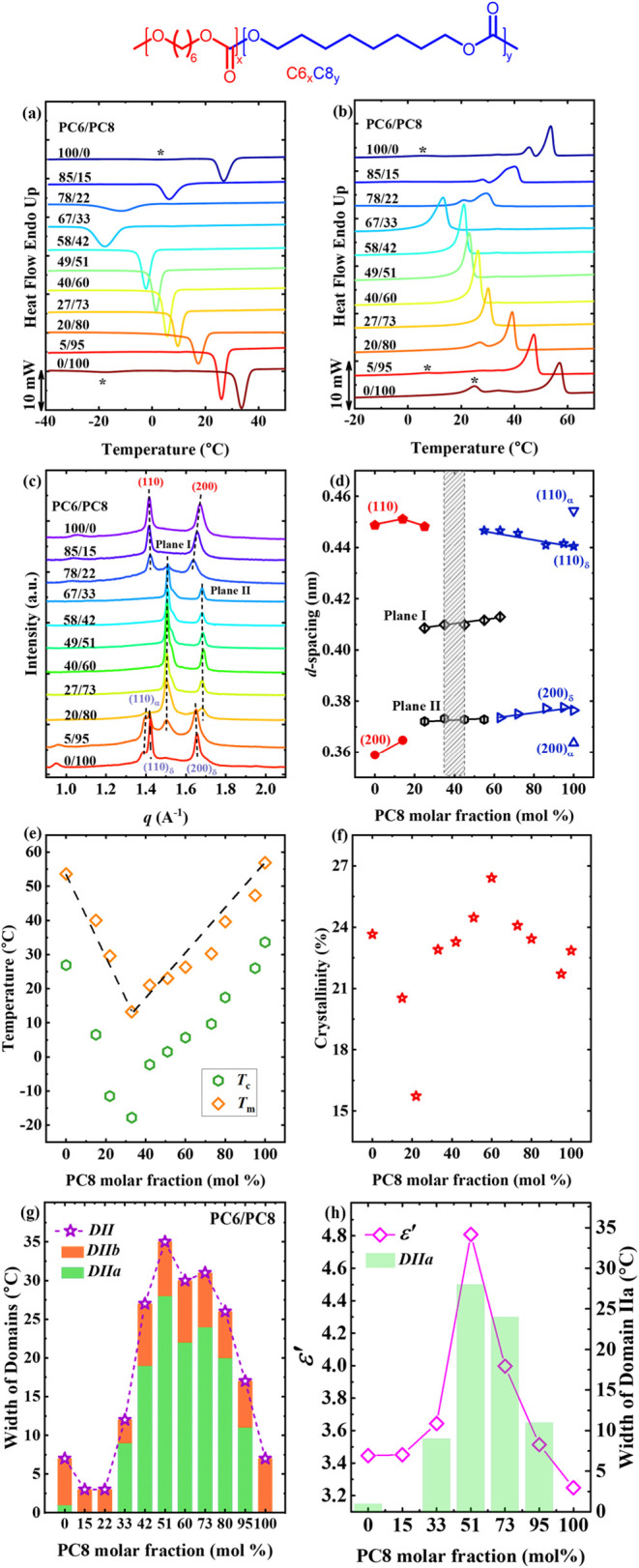
Summary of the evidence for mixed isodimorphic/isomorphic behavior in C6_*x*_C8_*y*_, including DSC (a) cooling and (b) second heating scans; (c) WAXS patterns, (d) *d*-spacing variation, (e) pseudoeutectic behavior in thermal transitions, and (f) *X*_c_; and the anomalous (g) SN and (h) dielectric responses associated with the γ-phase window. The C6_*x*_C8_*y*_ chemical structure is illustrated at the top. Figure 2 is adapted with permission from refs 5, 6, and 8. Copyright 2023, 2024, and 2026 American Chemical Society.

Follow-up studies on C7_*x*_C8_*y*_ and C12_*x*_C8_*y*_ copolymers^6,8^ demonstrated that this behavior is part of a broader pattern within the PC8-based family. More importantly, a clear structural trend is evident throughout the entire PC8-based copolycarbonate family. As the chain length of the second comonomer increases, the range of compositions where the γ phase forms becomes narrower. In C6_*x*_C8_*y*_, the γ phase exists across a wide composition range; in C7_*x*_C8_*y*_, this range is significantly smaller; and in C12_*x*_C8_*y*_, the γ phase only appears at a single composition, coexisting with PC8-type crystals. These results indicate that the stability of the isomorphic phase depends on the structural compatibility between the two repeating units. As the difference in methylene sequence length (*n*_CH_2__) increases, maintaining conformational matching and intermolecular packing within a shared crystal lattice becomes more difficult. Consequently, the energetic cost of fitting both repeat units into the same lattice increases, destabilizing the γ phase and narrowing its compositional stability window.

Additional insight into this phenomenon was gained from SN experiments (see Figure 2g,h), which revealed an unusual change in the self-nucleation *Domain* or *Domain II* within the isomorphic composition window (more details of this behavior can be obtained from reference 8). In classical isodimorphic copolymers, *Domain II* usually decreases as comonomer content increases.^4,20^ In contrast, the γ-phase region of these copolycarbonates, exhibits an expansion of *Domain II*, indicating an enhanced ability to preserve melt memory associated with ordered chain conformations. This behavior was linked to a partial restoration of intermolecular interactions in the isomorphic phase, as supported by dielectric (Figure 2h) and FT-IR measurements indicating stronger dipole-dipole interactions within the γ lattice.^8^

An even more complex situation was later reported in poly (butylene carbonate-*ran*-PC8) (C4_*x*_C8_*y*_) copolymers, where a triple mixed-mode involving isodimorphism, isomorphism, and total comonomer exclusion was identified within a single copolymer family,^7^ with its main features shown in Figure 3. In this system, the overall melting behavior still follows a pseudoeutectic trend, yet three distinct structural regimes can be distinguished: isodimorphic crystallization at low PC4 contents, formation of the γ phase in an intermediate compositional window, and total comonomer exclusion at high PC4 contents.

**Figure 3:**
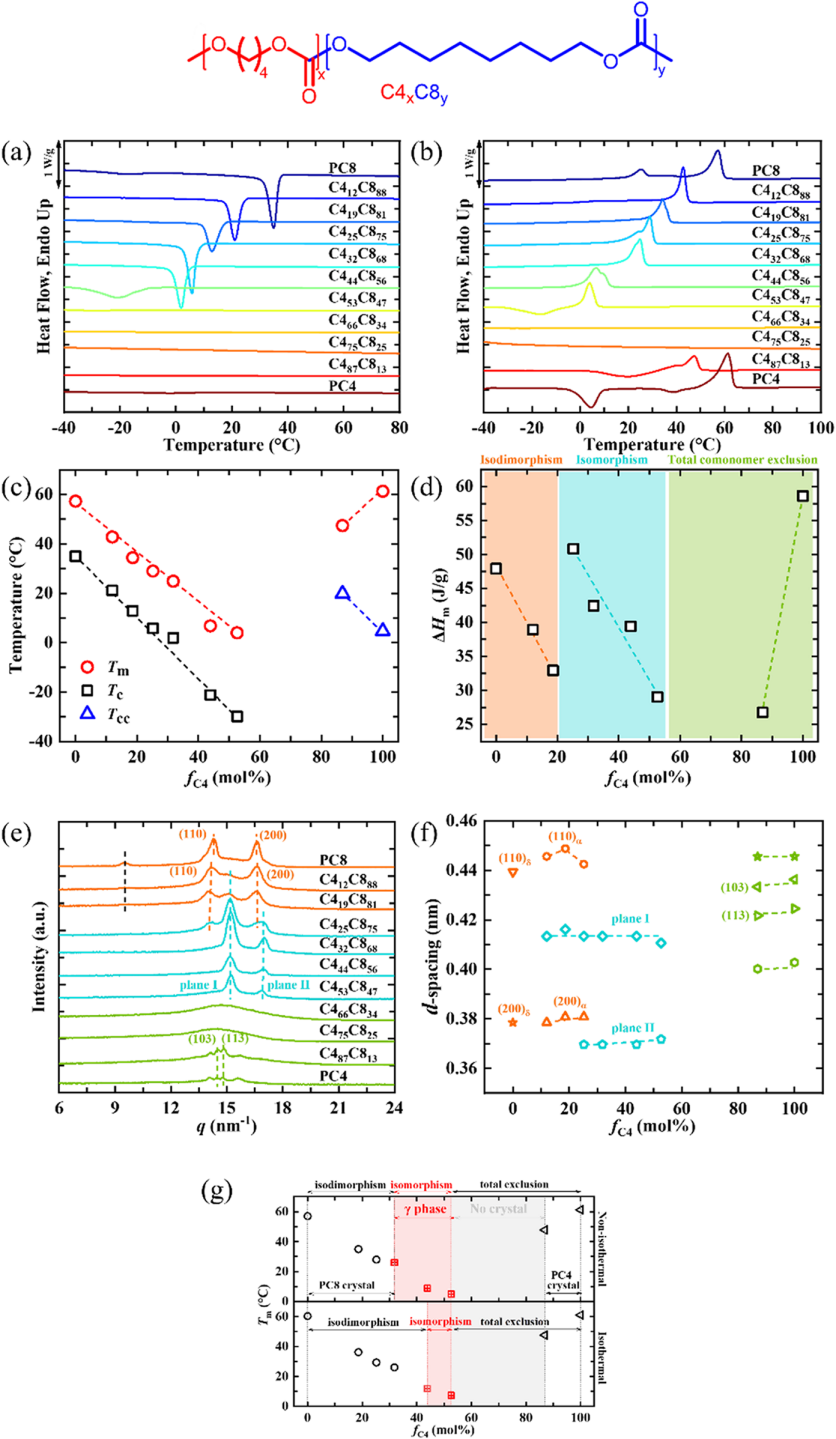
Representative example of triple mixed-mode behavior in C4_*x*_C8_*y*_ (chemical structure illustrated at the top), including DSC (a) cooling and (b) second heating scans; variation of (c) thermal transitions and (d) melting enthalpies as a function of comonomer content. (e) WAXS patterns, and (f) *d*-spacing vs comonomer content. In (d), the orange, cyan, and green shaded regions correspond to isodimorphism, mixed-mode crystallization involving the γ phase (isomorphism), and total comonomer exclusion, respectively, as indicated at the top of the panel. In (g), *T*_m_ vs comonomer content under nonisothermal (top panel) and isothermal (bottom panel) conditions is shown, indicating the dependence of the γ phase on crystallization conditions. Figure 3 illustrates how crystallization mode selection may become pathway-dependent, with nonisothermal and isothermal crystallization promoting different balances between comonomer inclusion and exclusion. Figure 3 is adapted with permission from ref 7. Copyright 2025 American Chemical Society.

Interestingly, the crystalline phase formed in the intermediate region depends heavily on crystallization conditions (Figure 3g). During nonisothermal cooling (Figure 3g, top panel), PC4 units may become kinetically trapped within the growing crystals, promoting the formation of the γ phase. In contrast, under isothermal crystallization (Figure 3g, bottom panel) at higher temperatures, increased chain mobility allows PC4 units to diffuse out of the crystalline lattice, favoring the formation of homopolymer-like crystals. These observations show that the balance between comonomer inclusion and exclusion is not solely thermodynamic but can also be strongly affected by crystallization kinetics.

Mixed crystallization behavior has also been observed in systems that display isodimorphism-exclusion transitions (Scheme 1c), such as poly­(ethylene succinate-*ran*-*ε*-caprolactone) (ES_*x*_CL_*y*_),^9^ and more recently in poly (propylene adipate-*ran*-butylene adipate) (PA_*x*_BA_*y*_) copolymers.^10^ In these materials, the *T*_m_ still follows a pseudoeutectic-like trend, but structural analysis shows that on one side of the composition range, the crystal lattice remains largely unchanged, indicating that the comonomer units are excluded rather than incorporated as defects. Furthermore, other features of this behavior were observed in PA_*x*_BA_*y*_ copolymers, especially in the composition region dominated by total comonomer exclusion. These include: a notable decrease in *X*_c_, slower crystallization kinetics, challenges in fractionation due to reduced *X*_c_ and disrupted intermolecular interactions, and measurement of zero comonomer inclusion^10^ using the comonomer inclusion/exclusion models.^21–24^

These observations demonstrate that similar thermal signatures can correspond to different crystallization mechanisms. Therefore, assignment of crystallization modes cannot rely solely on thermal transitions. Instead, a combination of thermal analysis, structural characterization, and sequence-sensitive techniques such as SN and SSA is often required to determine whether comonomer units are incorporated into the crystal lattice, occur as defects, or are completely excluded.

More broadly, the discovery of mixed crystallization modes necessitates a conceptual shift in interpreting crystallization behavior in random copolymers. The key question is no longer whether a system fits neatly into a single crystallization category, but rather how the balance between inclusion and exclusion evolves with composition and the crystallization pathway. This perspective is particularly important for local isomorphism, where conventional criteria derived from classical isodimorphism may be insufficient.

## 4 Evolution of Experimental Criteria for Crystallization Modes in Random Copolymers

Early studies on the crystallization of random copolymers established the experimental framework traditionally used to identify isodimorphic crystallization. Continuous crystallization, pseudoeutectic thermal behavior, and parent-like but distorted crystal lattices became the classical criteria for assigning isodimorphic crystallization.^1–4^

Over time, however, the study of semicrystalline random copolymers has incorporated a broader set of experimental techniques that probe crystallization beyond melting behavior alone. In addition to traditional nonisothermal DSC measurements, crystallization kinetics (Scheme 2a,2b) under controlled isothermal conditions^25,26^ have been widely used to investigate the development of nucleation rates, crystal growth rates, and overall crystallization kinetics^27–29^ as a function of composition.^25,26,30^ Equilibrium melting temperatures, *T*_m_^°^, obtained through Hoffman-Weeks analysis,^31^ along with structural characterization by techniques such as WAXS and SAXS, have further expanded the structural and thermodynamic description of these systems.

**Scheme 2:**
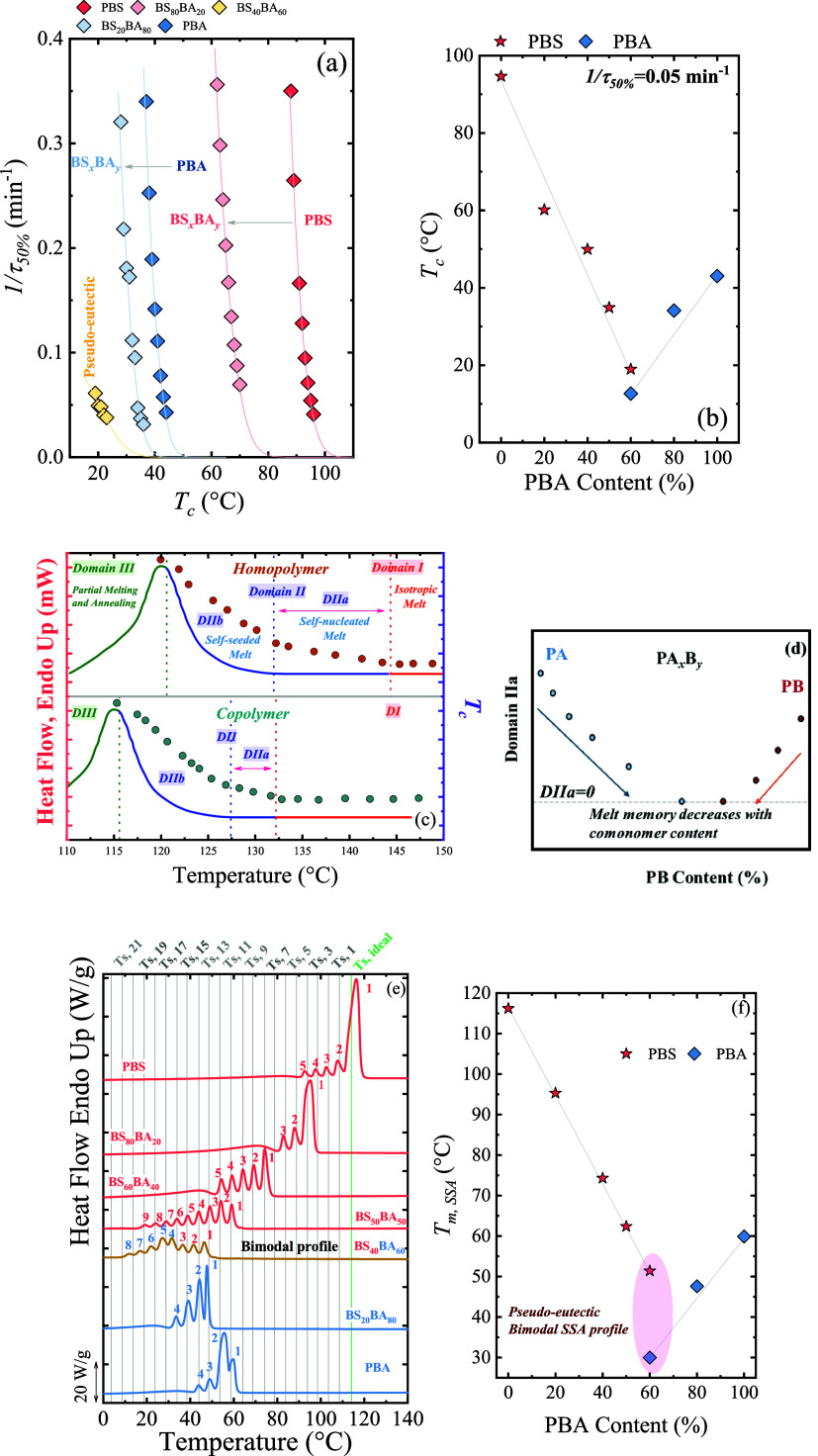
Schematic Representation of Experimental Results beyond Non-Isothermal Test^a^

a (a) Crystallization kinetics and its (b) pseudo-eutectic behavior; (c) SN of homopolymers and copolymers where (d) melt memory (*Domain IIa*) in copolymers is reduced; (e) SSA showing various profiles including bimodal profiles at the pseudo-eutectic point, and (f) pseudo-eutectic behavior of the melting point of the highest fraction. In (a), the fitting lines correspond to the Lauritzen and Hoffman theory,^28,29^ that also provides information on the copolymer crystallization behavior. Scheme 2 illustrates how sequence-sensitive and kinetic techniques provide additional experimental signatures beyond DSC thermal trends, enabling discrimination among different crystallization mechanisms in random copolymers. The experimental data employed to construct these schematic representations were obtained from refs 38 and 39.

Sequence-sensitive techniques have further expanded the experimental toolbox for studying random copolymer crystallization. In particular, SN experiments (Scheme 2b,2c) offer insights into melt memory and the stability of self-nuclei,^32–34^ while SSA experiments (Scheme 2d,e) enable thermal fractionation of the material,^19,35–37^ revealing the distribution of crystallizable sequences along the polymer chains. Importantly, SSA may also act as an intermediate crystallization protocol between rapid and slow crystallization conditions, thereby modifying the balance between comonomer inclusion and exclusion.^20,38^

These techniques have proven especially useful for detecting additional experimental signatures related to isodimorphic crystallization. In SN experiments, comonomer units incorporated as defects progressively weaken the intermolecular interactions that drive melt memory,^4,20,38,40^ as illustrated in Scheme 2d. As a result, the stability of self-nuclei decreases with increasing comonomer content, leading to a gradual reduction or even complete loss of melt memory. This monotonic weakening of melt memory can therefore be regarded as an additional signature of classical isodimorphic crystallization in polar copolymers.

SSA experiments provide complementary information about the distribution of crystallizable sequence lengths. In isodimorphic copolymers, excluded comonomer units disrupt crystallizable sequences, causing a decrease in the *T*_m_ of the highest SSA fraction (*T*_m,SSA_) as the comonomer content rises (Scheme 2e,2f). This disruption also tends to produce sharper and more clearly resolved fractionation profiles compared with those of the parent homopolymers.^20,38^ Near the pseudoeutectic composition, bimodal SSA profiles may emerge, reflecting coexistence of fractions associated with the two parent-like crystal structures.^38^

Additional experimental observables may also indicate the pseudoeutectic behavior typical of isodimorphic systems. For instance, nucleation rates, crystal growth rates, and overall crystallization kinetics often follow pseudoeutectic patterns based on composition (Scheme 2b). Likewise, properties that strongly depend on *X*_c_, such as the elastic modulus, frequently exhibit trends similar to those seen in *X*_c_, while elongation at break may display an inverted V-shaped pattern with a peak near the pseudoeutectic composition. Thus, isodimorphic crystallization influences not only melting behavior and diffraction patterns, but also broader kinetic and property trends.

The discovery of mixed crystallization modes has substantially changed how these criteria should be interpreted. In several systems, thermal measurements alone initially suggested classical isodimorphism because crystallization was maintained across the full composition range and *T*_m_ followed a pseudoeutectic dependence. However, more detailed analyses revealed localized deviations in Δ*H*_m_, *X*_c_, and diffraction behavior that coincided with the appearance of new crystalline phases distinct from the parent lattices.^5–8^ These observations showed that pseudoeutectic thermal behavior does not necessarily imply uniform isodimorphism across the entire composition range.

These local compositional windows demonstrate that the same global thermal signature may arise from different local crystallization mechanisms. In regions where a new common lattice forms, SN experiments reveal that melt memory may locally recover rather than decrease monotonically, indicating partial restoration of intermolecular interactions within the new phase.^8^ Conversely, systems approaching total comonomer exclusion exhibit disappearance of crystallization at intermediate compositions together with little or no variation in the unit-cell parameters of the parent crystals. These systems also display sharp reductions in crystallinity and associated thermal and mechanical performance.^9,10^

Collectively, these developments show that crystallization modes in random copolymers should no longer be assigned from a single level of evidence. Thermal behavior provides the initial phenomenological classification, whereas structural analysis determines whether the crystalline phase remains parent-like or becomes genuinely new. Sequence-sensitive techniques such as SN and SSA add a third level of information by revealing how comonomer incorporation affects melt memory and crystallizable sequence distributions.

From this perspective, the experimental challenge is not merely classificatory but diagnostic. This hierarchy of evidence is especially important in systems exhibiting local isomorphism or exclusion-dominated regions, where it helps distinguish true isomorphic crystallization from asymmetric isodimorphism or mixed-mode behavior. The next section applies these criteria to representative systems historically described as isomorphic.

## 5 Isomorphic Crystallization in Random Copolymers: Structural Criteria, Classification Limits, and Reassessment of Reported Systems

Isomorphic crystallization is the extreme case of complete comonomer inclusion in random copolymers, where both comonomer units are incorporated into a single crystalline lattice throughout the entire composition range. In these systems, crystallization occurs without forming separate parent-like crystalline phases, and the lattice parameters change smoothly as the copolymer composition varies.

In practice, however, the amount of experimental evidence supporting isomorphic crystallization varies significantly among reported systems. In this work, we distinguish three situations: (i) *strict isomorphism*, where a single crystalline phase distinct from the parent components and their known polymorphs is demonstrated across the full composition range; (ii) *limited-range isomorphism*, where shared-lattice behavior is observed only within a restricted compositional interval; and (iii) *isomorphic-like behavior*, where thermal and structural trends suggest isomorphism, but definitive proof of a genuinely shared crystalline lattice remains lacking. The main distinguishing features of these categories are summarized in Table 1.

**Table 1:** Working Criteria for Interpreting Reported Isomorphic Crystallization Behavior in Random Copolymers.

category	main characteristics	minimum supporting evidence
*Strict isomorphism*	Common (shared) crystalline lattice across the full composition range	New crystalline phase distinct from parent components and their known polymorphs, with systematic lattice evolution with composition
*Limited-range isomorphism*	Cocrystallization restricted to a specific compositional interval	Evidence of shared-lattice behavior only within a localized composition window
*Isomorphic-like behavior*	Thermal/structural trends suggest isomorphism, but proof is incomplete	DSC/WAXS trends compatible with isomorphism, but without clear evidence of a genuinely new common lattice

Importantly, these categories are not intended as rigid reclassification labels but rather as an evidence-based framework for interpreting the varying levels of structural support for reported isomorphic crystallization behavior in random copolymers.

Among the systems reported so far, only a few provide convincing evidence of strict isomorphic crystallization. One of the clearest examples is found in poly­(oxyhexamethylene-*ran*-oxydodecamethylene) (O6_*x*_O12_*y*_) copolyethers (Figure 4a),^41^ where crystallization occurs across the entire composition range (Figure 4b) while maintaining a single (new) crystalline phase (Figure 4c) with continuously changing lattice parameters (Figure 4d). The wide compositional coverage and consistent structural changes support the strength of this conclusion.

**Figure 4:**
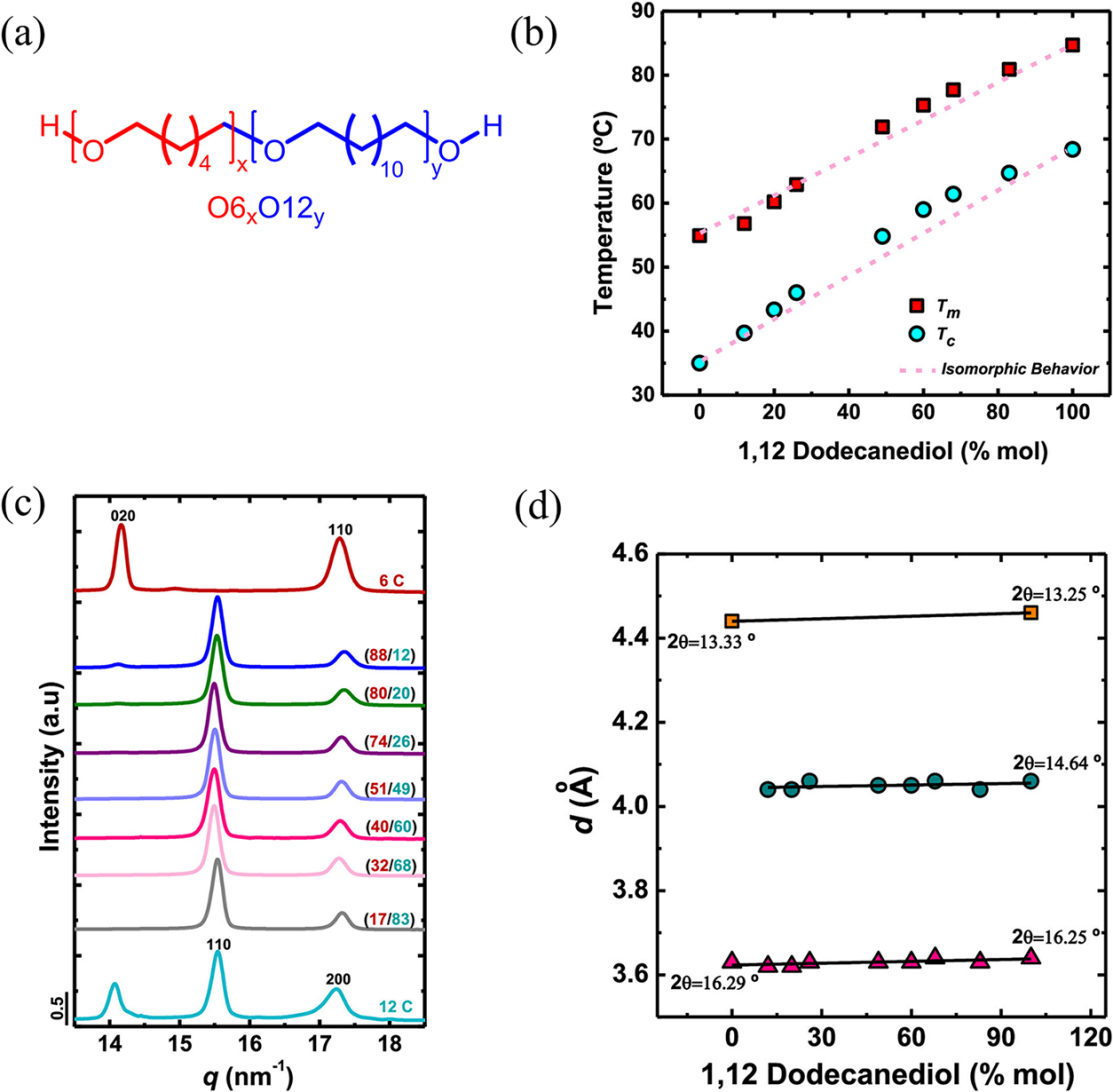
Representative strict isomorphism in O6_*x*_O12_*y*_ showing broad compositional coverage: (a) chemical structure , (b) continuous *T*_m_ evolution, and (c, d) systematic lattice evolution by WAXS. Figure 4 illustrates one of the clearest reported examples of *strict isomorphism*, in which continuous thermal evolution is accompanied by systematic structural evolution within a single crystalline phase across the entire composition range. Figure 4 is adapted with permission from ref 41. Copyright 2019 American Chemical Society.

Several other systems have been described as isomorphic, primarily based on thermal behavior and X-ray diffraction patterns. For example, poly­(butylene succinate-*ran*-butylene fumarate) (BS_*x*_BF_*y*_) copolyesters (Figure 5a)^42^ show nearly linear *T*_m_ trends with composition (Figure 5b), similar WAXS patterns (Figure 5c), and comparable *d*-spacing (Figure 5f). However, limited compositional resolution and a lack of clear structural evidence for a new crystalline lattice suggest these systems are better classified as *isomorphic-like* rather than exhibiting *strict isomorphism*. For instance, in Figure 5c, the WAXS patterns of the PBF- and BF-rich compositions resemble those of PBF, and a mirrored situation occurs for the PBS- and BS-rich compositions. Despite this, claims of isomorphic behavior have been made for PBS/PBF blends^43^ and a terpolymer formed by PBF–PBS-PBA.^42,43^

**Figure 5:**
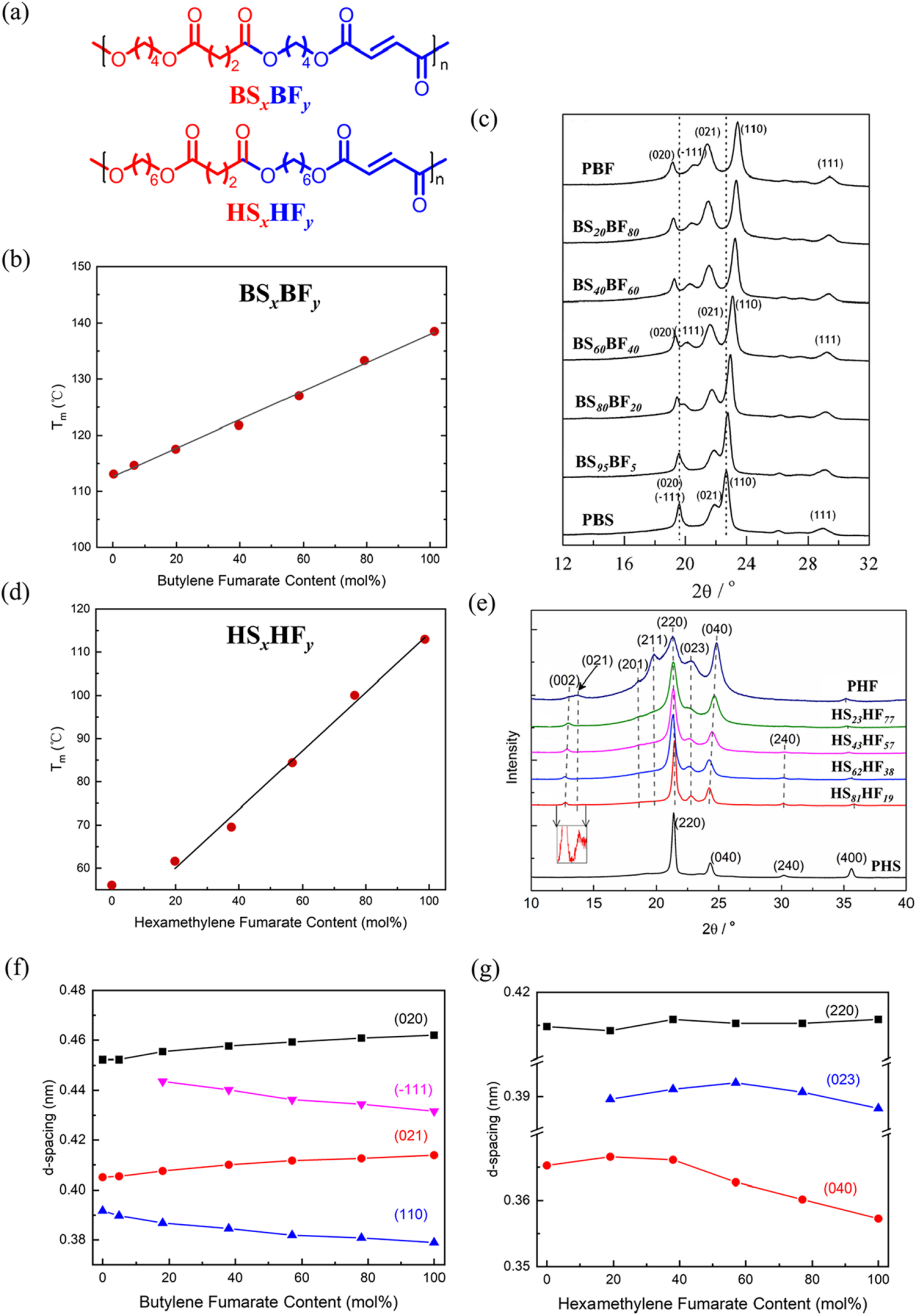
Examples of isomorphic-like behavior in BS_*x*_BF_*y*_ and HS_*x*_HF_*y*_ chemical structures in (a). The *T*_m_ (b, d) and *d*-spacing (f, g) vs comonomer content is shown for BS_*x*_BF_*y*_ (b, f) and HS_*x*_HF_*y*_ (d, g), and the WAXS pattern is shown in (c) BS_*x*_BF_*y*_ and (e) HS_*x*_HF_*y*_. In both systems, thermal continuity alone is insufficient to conclusively demonstrate *strict isomorphism*, as a distinct common crystalline phase separate from the parent lattices and their known polymorphs is not clearly observed. Figure 5 is adapted from refs 44 and 45. Copyright 2012 American Chemical Society and Copyright 2014 Elsevier.

In other cases, isomorphic crystallization is limited to specific compositional ranges, although cocrystallization was not claimed. Poly­(hexamethylene succinate-*ran*-hexamethylene fumarate) copolyesters (HS_*x*_HF_*y*_)^45^ (Figure 5a) provide a representative example (Figure 5d,5e,5g), where isomorphic behavior or a coexistence between PHF and PHS phases over a wide composition range is observed only in fumarate-rich compositions (Figure 5e). This suggests that comonomer inclusion is favored only within a localized compositional range, while other parts of the composition spectrum deviate from strict isomorphism. Accordingly, these systems are better described as exhibiting *limited-range isomorphism* rather than *strict isomorphism*. However, broader compositional and structural analyses would still be necessary to determine whether mixed crystallization behavior may also exist outside the currently studied composition range.

Although mixed crystallization modes have so far been most clearly identified in a limited number of systems, similar behaviors may already be present in additional random copolymer families but remain underrecognized because of the historical tendency to classify crystallization behavior exclusively within the classical isomorphism/isodimorphism/exclusion framework. Additional studies combining broad compositional sampling with structural and sequence-sensitive characterization will be necessary to clarify the generality and limits of these mixed crystallization regimes.

An additional complication occurs in systems where the parent homopolymers already have very similar crystal structures. In copolyesters such as poly­(hexamethylene glutarate-*ran*-hexamethylene azelate), HG_*x*_HAz_*y*_ ^46^ (Figure 6a), continuous lattice variations (Figure 6d) do not necessarily imply the formation of a new crystalline phase because both components may already share closely related packing arrangements (Figure 6b). In such cases, distinguishing true isomorphism from structural similarity requires more detailed experimental analysis.

**Figure 6:**
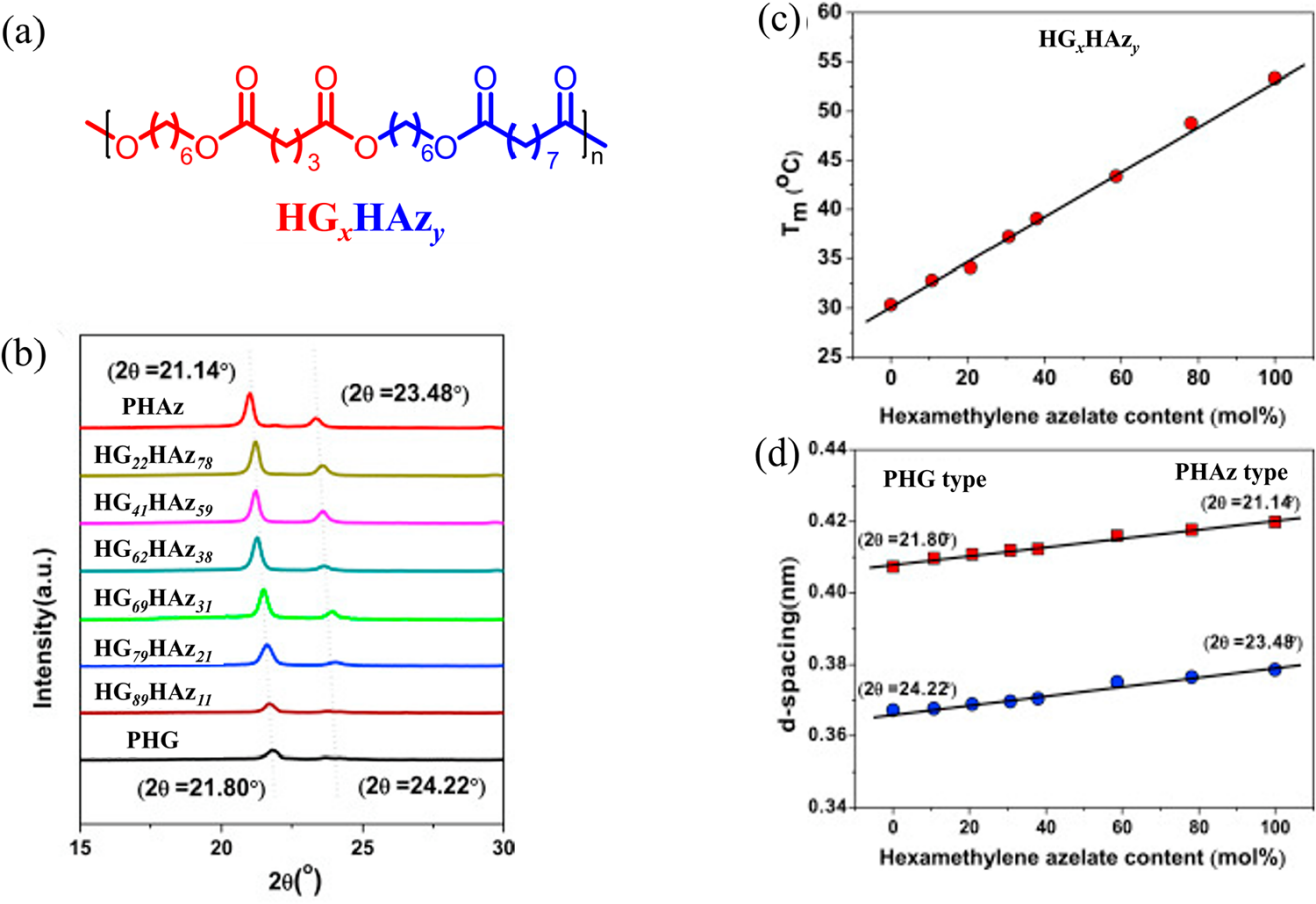
Systems with highly similar parent lattices. (a) HS_*x*_HAz_*y*_ chemical structure, (b) WAXS patterns, (c) *T*_m_ vs comonomer content evolution, and (d) *d*-spacing variation with comonomer content. Figure 6 illustrates how strong structural similarity between parent lattices may complicate the identification of genuinely new common crystalline phases. Figure 6 is adapted from ref 46. Copyright 2017 Elsevier.

Importantly, a key aspect that emerges from analyzing these systems is the role of compositional sampling. While some studies examine a sufficiently dense set of compositions, others depend on a limited number of samples, which may hide important features such as pseudoeutectic behavior or transitions between crystallization modes.

A particularly illustrative example is provided by poly­(ω-pentadecalactone-*ran*-PCL), PDL_*x*_CL_*y*_ (Figure 7a) copolyesters.^20,47,48^ In early studies,^47,48^ a limited number of compositions yielded a linear variation in *T*_m_, suggesting isomorphic crystallization. However, subsequent investigations with expanded compositional coverage revealed highly asymmetric pseudoeutectic behavior, demonstrating that the system is, in fact, isodimorphic.^20^ This example, illustrated in Figure 7b,7c, shows that the absence of a detectable pseudoeutectic minimum does not necessarily indicate isomorphism; it may instead result from insufficient compositional resolution.

**Figure 7:**
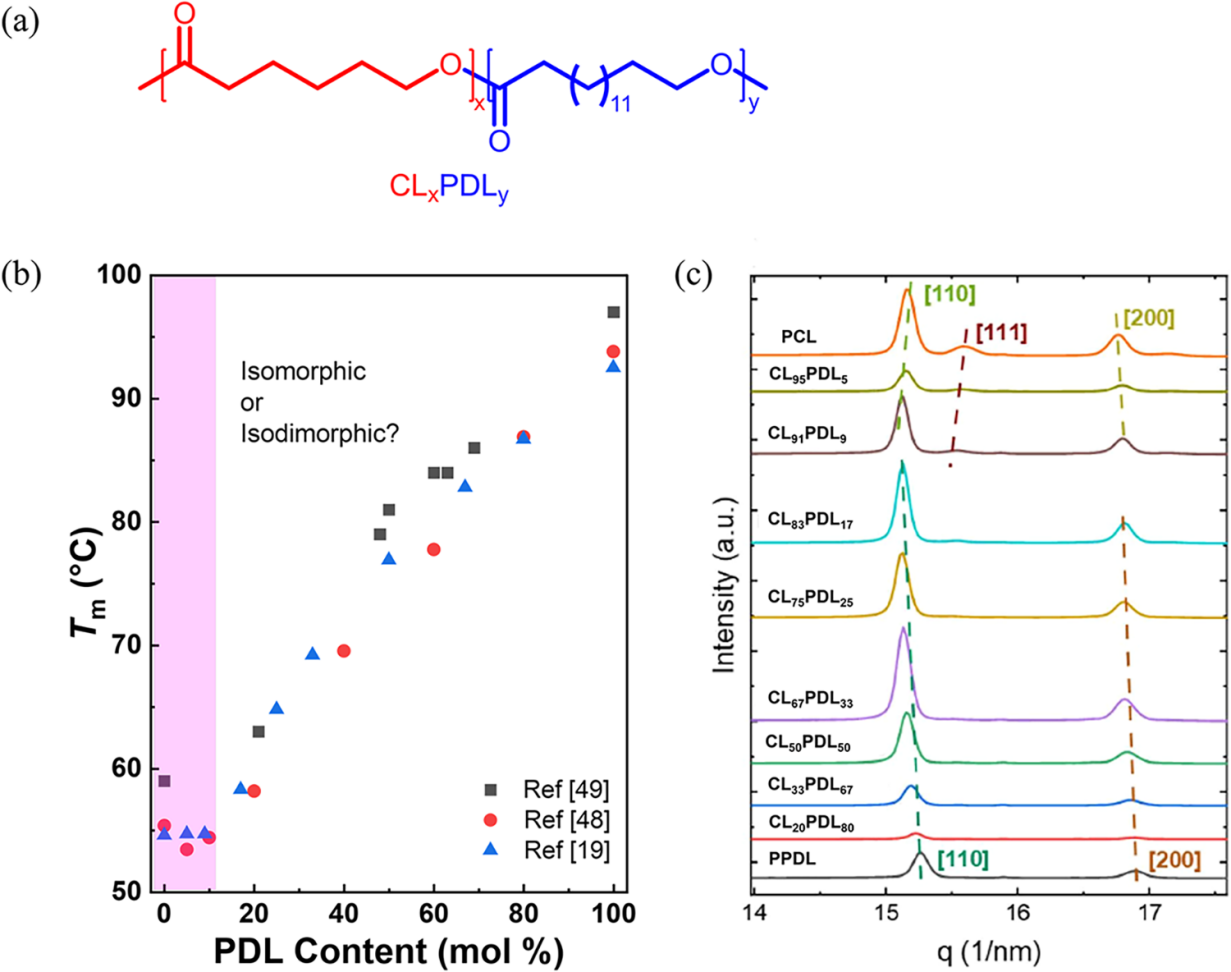
(a) PDL_*x*_CL_*y*_ chemical structure, (b) comparison of *T*_m_ vs PDL content of works reported in the literature,^20,47,48^ and (c) WAXS patterns. The shaded region in (b) highlights the compositional interval in which the asymmetric pseudoeutectic behavior, and thus the isodimorphic character of the system, becomes evident. Figure 7 highlights how insufficient compositional sampling may conceal pseudoeutectic behavior and lead to overestimation of *strict isomorphic* crystallization. Figure 7 is adapted from refs 47, 48, and 20. Copyright 2015, 2005, and 2025 American Chemical Society.

More generally, three main sources of ambiguity can be identified in the literature: (i) insufficient compositional sampling, which may conceal pseudoeutectic behavior; (ii) inherent structural similarity between parent homopolymers, making it harder to distinguish a separate crystalline phase; and (iii) limited experimental evidence, especially when kinetic or sequence-sensitive techniques do not support structural characterization. These limitations may lead to overestimation of *strict isomorphism* when structural assignment relies predominantly on DSC continuity.

Furthermore, systems exhibiting *partial isomorphism* may, in some cases, correspond to mixed crystallization modes, in which compositional windows of cocrystallization coexist with regions dominated by isodimorphic crystallization or comonomer exclusion.

Overall, this reassessment indicates that strict isomorphic crystallization is less common than often assumed. Many systems historically labeled as isomorphic are better described as exhibiting *limited-range isomorphism*, asymmetric isodimorphism, or potentially mixed crystallization modes when broader compositional and structural analyses are considered. Therefore, convincing identification of *strict isomorphism* requires more than thermal continuity alone. Ideally, it should combine sufficient compositional resolution, structural evidence distinguishing a genuinely new common lattice from parent-like phases or known polymorphs, and complementary sequence-sensitive or melt-memory-related analyses. Confidence in crystallization-mode assignment therefore depends on the convergence of thermal, structural, compositional, and sequence-sensitive evidence rather than on any single experimental descriptor. The rarity of strict isomorphism does not diminish its importance. On the contrary, it shows that true common-lattice crystallization in random copolymers requires a very narrow balance of geometric compatibility, conformational flexibility, and intermolecular interactions. Most systems do not satisfy these conditions globally and instead occupy intermediate regimes. This refined framework provides the basis for the comparative analysis of polymer families developed in the next section.

## 6 Structural Insights from Isodimorphic Crystallization across Polymer Families

Isodimorphic crystallization is typically indicated by a pseudoeutectic minimum in *T*_m_-composition diagrams. However, this feature alone does not fully capture the diversity of crystallization behaviors observed in random copolymers. A more informative approach is to compare polymer families using the structural variables that govern defect accommodation, lattice competition, and access to mixed crystallization modes.

The pseudoeutectic point indicates the composition where competition between two parent-like crystalline phases is at its peak. When this point is near an equimolar composition, the system can be described as pseudoeutectically symmetric, showing that both crystalline lattices have a similar ability to accommodate defects. Conversely, strongly shifted pseudoeutectic compositions indicate pseudoeutectic asymmetry, which reflects a preference for one comonomer to be included into the lattice of the other.

Importantly, pseudoeutectic symmetry does not directly measure the extent of inclusion. Symmetric systems may still show limited mutual incorporation, while highly asymmetric systems might indicate efficient but strongly directional defect accommodation. Instead, pseudoeutectic position reflects the combined effects of geometric compatibility, conformational constraints, intermolecular interactions, polymorphism, and crystallization kinetics.

From this perspective, the value of comparing polymer families is not simply to multiply examples, but to identify which structural parameters dominate in each case. In aliphatic copolyesters, crystallization behavior is mainly tuned by repeat-unit periodicity, ester-group density, and conformational adaptability. In copolycarbonates, the balance shifts toward methylene-sequence-dependent packing, even-odd effects, and the appearance of additional crystalline phases. In polyamides, hydrogen-bond periodicity and polymorphic accessibility add an additional level of constraint.

The goal of this section is not simply to restate that many systems are isodimorphic, but rather to explain why similar thermal signatures may emerge from very different structural balances. These balances ultimately determine the boundaries between isodimorphism, isomorphism, exclusion, and mixed crystallization behavior.

### 6.1 What Aliphatic Polyesters Reveal: Multiple Structural Routes to Isodimorphism

Aliphatic polyesters provide a particularly versatile framework for studying crystallization in random copolymers because their molecular architecture can be systematically modified through multiple synthetic routes. This flexibility enables controlled variation of chain periodicity, ester-group density, and conformational adaptability, allowing direct evaluation of how subtle structural changes influence packing efficiency, defect accommodation, and the balance between comonomer inclusion and exclusion.

As a consequence, aliphatic polyesters make it possible to isolate how small changes in molecular architecture alter packing efficiency, defect tolerance, and the balance between comonomer inclusion and exclusion.

Within this family, three complementary subspaces are particularly informative: diol/diacid systems, which are ideal for examining repeat-unit periodicity and host-guest asymmetry; polylactones, which reveal the role of ester density within a chemically uniform framework; and hybrid systems, which expose the consequences of combining distinct repeat-unit architectures within the same chain.

#### 6.1.1 Diol/Diacid-Based Copolyesters: Structural Similarity, Asymmetry, Conformational Constraints, and Lattice Adaptability

Diol/diacid-based copolyesters serve as the most versatile platform for analyzing crystallization in semicrystalline polymers because both segments of the repeat unit can be modified independently. This synthetic flexibility allows systematic variation in methylene sequence length (*n*_CH_2__), the distance between ester groups, and the overall periodicity of the repeat unit. As a result, it is possible to transition seamlessly from systems that are nearly isomorphic to strongly asymmetric isodimorphic systems, and finally to cases approaching total exclusion. Consequently, these materials provide a unique framework for understanding how small versus large structural changes influence pseudoeutectic behavior.

##### 6.1.1.1 Systems Approaching Isomorphism: Conformational Compatibility as a Limiting Condition

The upper limit of compatibility in diol/diacid copolyesters is represented by systems often described as nearly isomorphic. In these cases, *T*_m_s may vary approximately linearly with composition; however, as discussed in the previous section, such thermal behavior alone is insufficient to demonstrate true isomorphism. The key question is not whether the thermal response appears continuous, but whether a genuinely new common lattice is formed.

A representative example is BS_*x*_BF_*y*_ ^44^ (Figure 5a), where the diol segment is identical in both repeat units, while the diacid segments contain the same total number of carbon atoms (four). The only difference is the presence of a double bond in the fumarate unit, whereas succinate contains a saturated −CH_2_–CH_2_– segment. Despite this small chemical difference, the *trans* configuration of the fumarate unit maintains a linear geometry compatible with the all-*trans* conformation of the PBS chain. As a result, the spacing between ester groups and the overall chain conformation remain sufficiently similar to allow both repeat units to pack within a common lattice, leading to isomorphic-like behavior (Table 1) rather than unequivocal strict isomorphism (Figure 5).

This apparent compatibility, however, is highly sensitive to local geometry. When the fumarate unit is replaced by its *cis* isomer, maleate (PBM), while keeping the same diol component and the same number of carbons in the diacid segment, the crystallization behavior changes significantly. In BF_*x*_BM_*y*_,^49^ the *cis* double bond creates a pronounced kink in the chain that disrupts conformational matching and breaks the translational periodicity needed for crystal packing. Consequently, crystallization is no longer supported by a common lattice; instead, it depends on fumarate-rich sequences, while maleate units are mostly excluded from the crystalline regions and act as randomly distributed defects that limit the length of crystallizable sequences. These observations demonstrate that the relevant criterion is not nominal chemical similarity, but local geometric compatibility.

A more structurally complex example is found in HS_*x*_HF_*y*_ ^45^ (Figure 5a), which can be directly compared with BS_*x*_BF_*y*_ ^44^ (Figure 5a), because both systems maintain the same diacid chemistry, containing the same number of carbon atoms and differing only by a *trans* double bond in the fumarate unit, while varying the length of the diol segment. This makes them an ideal platform for isolating the effect of chain architecture on crystallization behavior. In HS_*x*_HF_*y*_ copolymers, a behavior consistent with *limited-range isomorphism* (Table 1) has been reported despite significant changes in chain conformation and crystal structure. These observations suggest that the apparent thermal continuity arises from structural reorganization and polymorphic competition rather than from genuine cocrystallization within a common lattice.

These observations highlight that isomorphism should not be inferred from thermal continuity alone. In diol/diacid polyesters, the boundary between true isomorphism and isomorphic-like behavior is narrow and can be displaced by subtle local perturbations.

##### 6.1.1.2 Near-Symmetric Isodimorphism: Small Mismatch and Balanced Competition

When the structural similarity between comonomers slightly decreases, isomorphism or isomorphic-like behavior is lost, and isodimorphism appears, usually with a pseudoeutectic minimum near equimolar compositions. This behavior occurs in systems such as poly (hexamethylene succinate-*ran*-hexamethylene adipate), HS_*x*_HA_*y*_,^50^ where the diacid segments differ by only two methylene groups (succinate: 2 CH_2_, adipate: 4 CH_2_). Despite the similarity in the parent crystal structures and lattice parameters, the system shows isodimorphism rather than isomorphism, with a relatively shallow pseudoeutectic minimum typically close to 50–60 mol % of the longer component.

This shows that even small differences in (i) ester-group spacing, (ii) chain folding requirements, and (iii) conformational preferences are enough to prevent the formation of a common lattice. Near-symmetric pseudoeutectic behavior should not be interpreted as evidence of extensive mutual inclusion, but rather as balanced competition between two defect-tolerant parent-like lattices.

A similar situation occurs in poly­(butylene succinate-*ran*-butylene adipate), BS_*x*_BA_*y*_ ^38,51^ (Figure 8a), where the same diacid mismatch is introduced (succinate: 2 CH_2_; adipate: 4 CH_2_; Δ*n*_CH_2__ = 2), while the diol length remains constant. In this case, the pseudoeutectic minimum is also found near intermediate compositions, around 50–60 mol % (Figure 8b), and the system maintains a highly balanced competition between the two crystalline phases. However, BS_*x*_BA_*y*_ shows a stronger dependence on thermal history, including coincident crystallization, sequential melting, and rate-dependent transitions between single- and double-crystalline states.^38,51^ This demonstrates that pseudoeutectic symmetry does not imply mechanistic simplicity.

**Figure 8:**
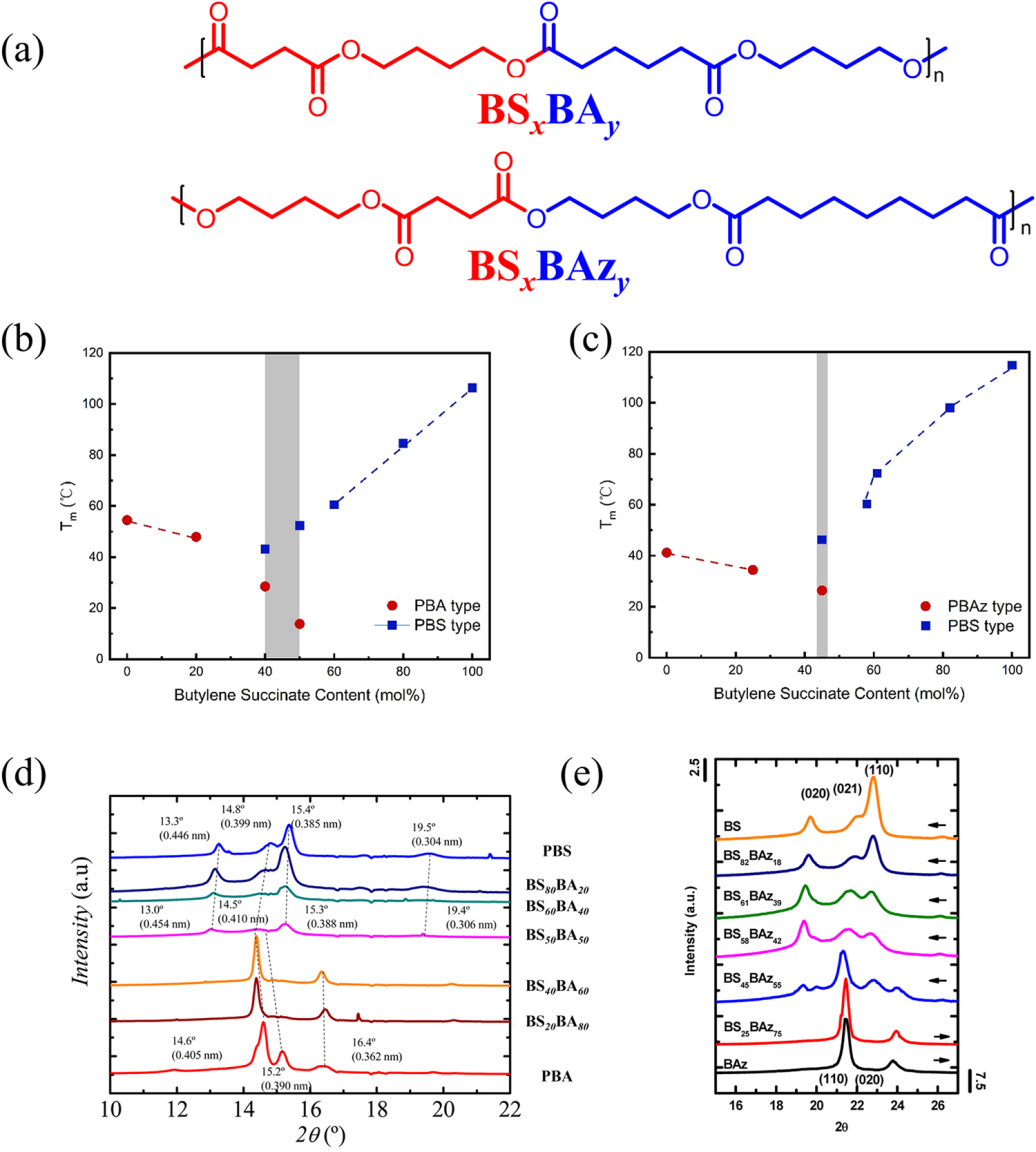
(a) Chemical structures of BS_*x*_BA_*y*_ and BS_*x*_BAz_*y*_; *T*_m_ vs comonomer content for (b) BS_*x*_BA_*y*_ and (c) BS_*x*_BAz_*y*_ copolymers. In (d, e), the WAXS patterns are shown for BS_*x*_BA_*y*_ and BS_*x*_BAz_*y*_ copolymers, respectively. Figure 8 illustrates how systems with similar thermal symmetry may nevertheless exhibit distinct sensitivities to thermal history and crystallization pathways. Figure 8 is adapted from refs 38,51, and 52. Copyright 2020, 2017, and 2015 American Chemical Society.

This behavior can be directly compared with poly­(butylene succinate-*ran*-butylene azelate), BS_*x*_BAz_*y*_,^52^ (Figure 8a), where the diol segment remains unchanged, and only the diacid length is further increased (succinate: 2 CH_2_; azelate: 7 CH_2_; Δ*n*_CH_2__ = 5). Despite this much larger mismatch, the system still displays isodimorphic behavior across the full composition range, with a pseudoeutectic minimum near 45–55 mol % (Figure 8c). This result is particularly important because it shows that increasing mismatch does not necessarily drive the system toward strong asymmetry or exclusion.

Related systems clearly show how difficult it is to create a simple rule based solely on methylene-unit content mismatch, especially when the diol segment remains the same and only the diacid length changes. Yu et al.^46^ studied a series including poly­(hexamethylene glutarate-*ran*-hexamethylene pimelate) (HG_*x*_HP_*y*_), poly­(hexamethylene pimelate-*ran*-hexamethylene azelate) (HP_*x*_HAz_*y*_), and HG_*x*_HAz_*y*_, where the diacid parts have 3 (HG), 5 (HP), and 7 (HAz) methylene units, respectively. Therefore, HG_*x*_HP_*y*_ and HP_*x*_HAz_*y*_ have a Δ*n*_CH_2__ of 2, while HG_*x*_HAz_*y*_ has a larger mismatch of 4 (Figure 9a).

**Figure 9:**
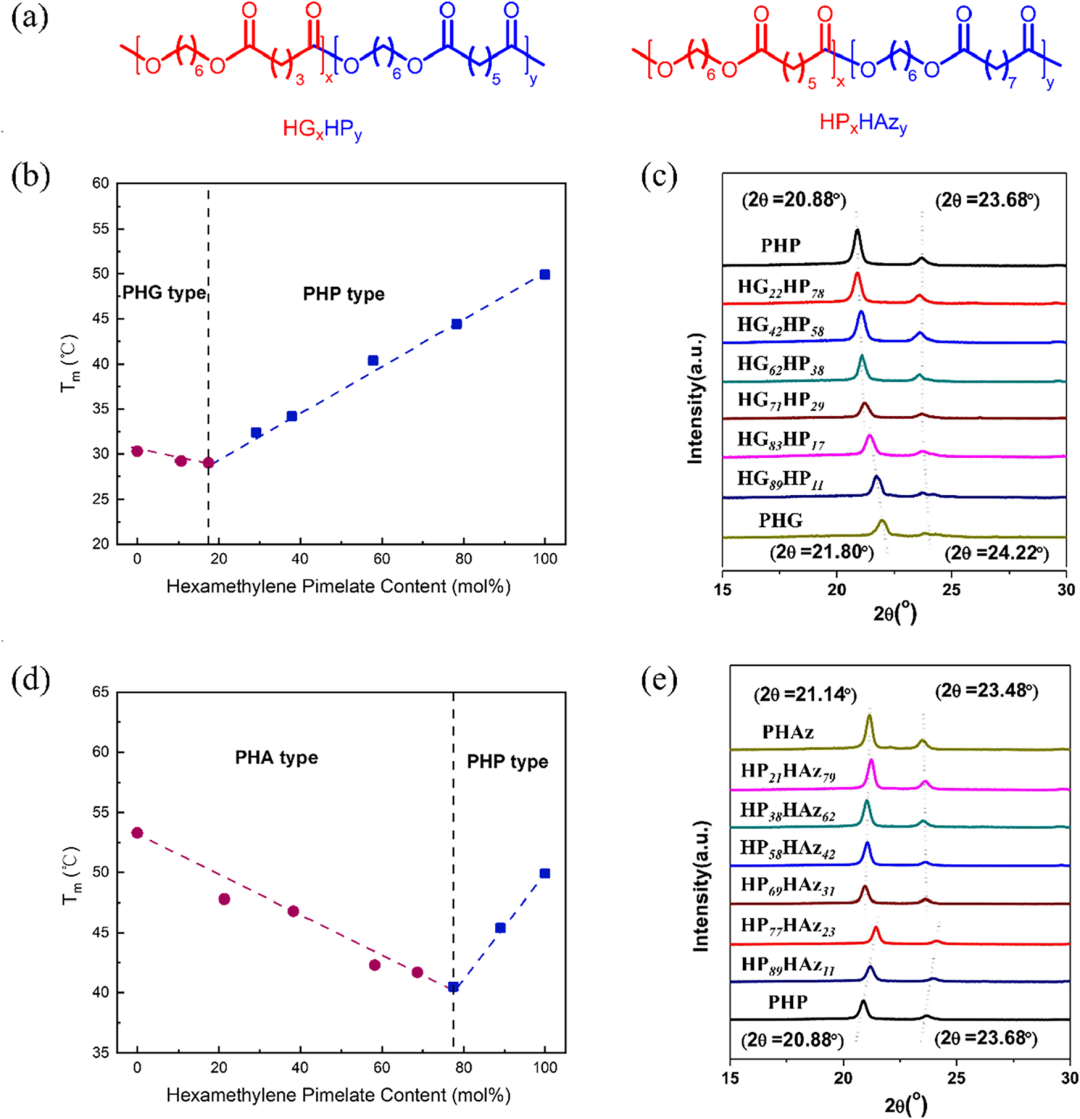
(a) Chemical structures for HG_*x*_HP_*y*_ and HP_*x*_HAz_*y*_ copolymers; *T*_m_ vs comonomer content for (b) HG_*x*_HP_*y*_ and (d) HP_*x*_HAz_*y*_ copolymers. The WAXS patterns for these copolymers are shown in (c) HG_*x*_HP_*y*_ and (e) HP_*x*_HAz_*y*_. Figure 9 is adapted from ref 46. Copyright 2017 Elsevier.

The Δ*n*_CH_2__ = 2 systems show clear isodimorphic behavior, with pseudoeutectic points significantly shifted toward the longer-chain component (∼80 mol %), indicating a preference for incorporating the shorter comonomer into the lattice of the longer one. This provides direct evidence of directional inclusion and asymmetric lattice adaptability.

In contrast, HG_*x*_HAz_*y*_ (Figure 6), despite having a larger mismatch, has been reported as isomorphic.^46^ This assignment should be approached with caution, as it primarily relies on trends in thermal properties and similarities in diffraction patterns rather than definitive evidence of a new common lattice. More importantly, when combined with the strongly asymmetric behavior of the Δ*n*_CH_2__ = 2 systems, this case shows that there is no universal, monotonic relationship between Δ*n*_CH_2__ and the crystallization mode.

These comparisons indicate that near-symmetric isodimorphism does not simply result from a small mismatch in methylene units, nor does a larger mismatch necessarily lead the system toward strong asymmetry or exclusion. Rather, pseudoeutectic symmetry reflects how the full repeat-unit architecture controls the relative competitiveness of the two lattices.

##### 6.1.1.3 Strongly Asymmetric Systems: Progressive Displacement of the Pseudoeutectic

As the difference in methylene sequence length grows, the pseudoeutectic point gradually shifts toward the longer-chain comonomer, indicating increased asymmetry in lattice competitiveness. This behavior is particularly well documented in the family of poly­(hexamethylene succinate)-based copolyesters (Figure 10a), where the diol segment remains constant (hexamethylene) and only the diacid length varies.^50^

**Figure 10:**
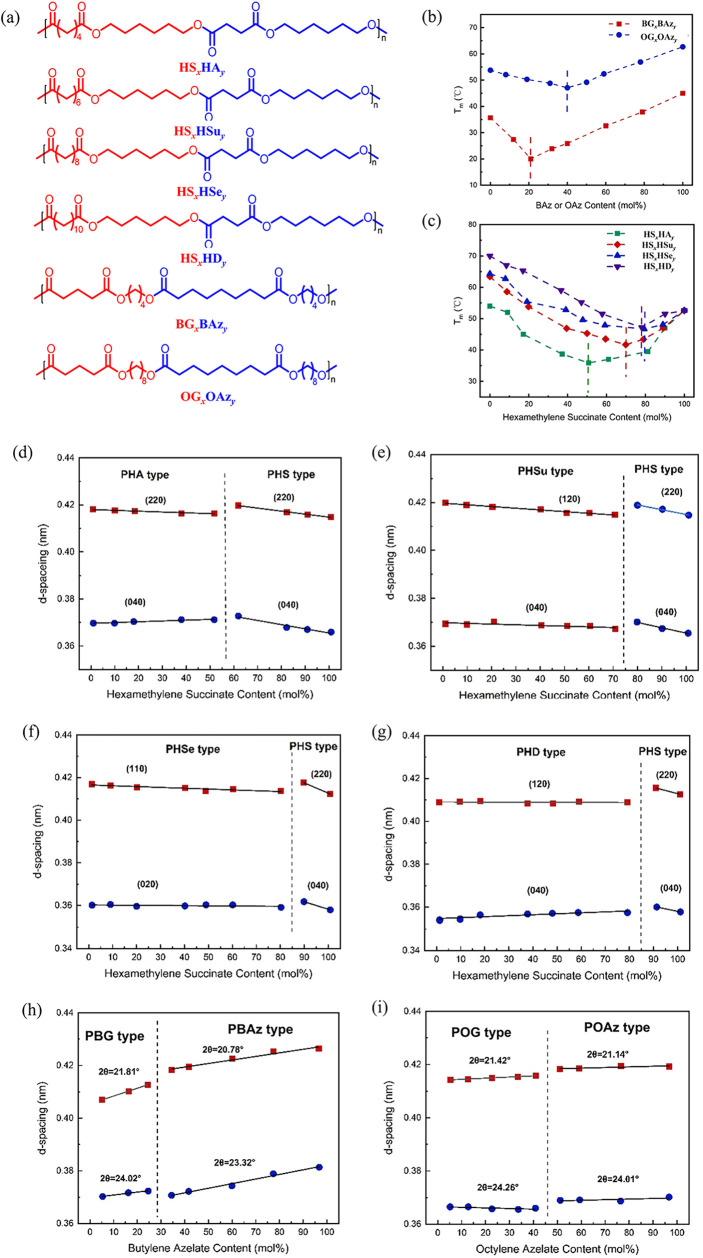
(a) Chemical structures for PHS-based copolymers and BG_*x*_BAz_*y*_ and OG_*x*_OAz_*y*_ copolymers. (b) Comparison of the shift in the pseudoeutectic point (in *T*_m_ vs comonomer content) for BG_*x*_BAz_*y*_ and OG_*x*_OAz_*y*_ copolymers. (c) Evolution of pseudoeutectic point with *n*_CH_2__ for PHS-based systems. (d–i) *d*-spacing variations for PHS-based systems. Figure 10 highlights how pseudoeutectic asymmetry evolves systematically with repeat-unit architecture and cannot be predicted solely from local methylene mismatch. Figure 10 is adapted from refs 50 and 53. Copyright 2017 and 2018 Elsevier.

In HS_*x*_HA_*y*_, the diacid mismatch is Δ*n*_CH_2__ = 2 (succinate: 2 CH_2_; adipate: 4 CH_2_), and the pseudoeutectic point is located near 50–55 mol % HS. As the diacid length increases, the pseudoeutectic shifts progressively toward HS-rich compositions: for suberate (HS_*x*_HSu_*y*_, 6 CH_2_; Δ*n*_CH_2__ = 4), it appears at ∼70–75 mol % HS; for sebacate (HS_*x*_HSe_*y*_, 8 CH_2_; Δ*n*_CH_2__ = 6), at ∼80–85 mol % HS; and for dodecanedioate HS_*x*_HD_*y*_, 10 CH_2_; Δ*n*_CH_2__ = 8, it remains in the range of ∼80–85 mol % HS, as shown in Figure 10c.

This systematic displacement indicates a gradual increase in the stability and defect tolerance of the longer-chain lattice. This asymmetry originates from the greater stability and defect tolerance of the longer-chain lattice. Longer polymethylene sequences reduce ester-group density and promote more PE-like conformations, thereby lowering the energetic penalty associated with incorporation of shorter units.

A similar asymmetric trend was previously observed in HG_*x*_HP_*y*_ and HP_*x*_HAz_*y*_ (Figure 9), where the pseudoeutectic points are already significantly shifted toward the longer-chain component, occurring at approximately 75–80 mol % HP and HAz, respectively.^46^

Notably, this strong asymmetry occurs even at a relatively small mismatch (Δ*n*_CH_2__ = 2), unlike the HS-based series, where a similar mismatch still results in near-symmetric behavior. This comparison reinforces the idea that the pseudoeutectic position is controlled not by mismatch alone, but by how that mismatch perturbs packing efficiency and defect tolerance in each competing lattice.

These systems show that the pseudoeutectic position is a clear experimental sign of directional inclusion, where one crystalline phase acts as a host lattice and the other as a defect species. As this asymmetry grows, the system moves further away from balanced isodimorphism and displays increasingly asymmetric crystallization behavior.

##### 6.1.1.4 Effective Repeat Unit Periodicity: Beyond Local Mismatch

Although a methylene mismatch in a single segment is clearly significant, it is not enough to predict the pseudoeutectic position. A more complete understanding arises when both segments of the repeat unit are considered together, as this influences the chain’s effective periodicity.

This is well illustrated by comparing poly­(butylene glutarate-*ran*-butylene azelate), BG_*x*_BAz_*y*_, and poly­(octylene glutarate-*ran*-octylene azelate), OG_*x*_OAz_*y*_ (Figure 10a), where the diacid pair is the same (glutarate: 3 CH_2_; azelate: 7 CH_2_; Δ*n*_CH_2__ = 4), but the diol varies from butylene: 4 CH_2_ to octylene: 8 CH_2_.^53^

Despite having the same diacid mismatch, the pseudoeutectic positions vary significantly: in BG_*x*_BAz_*y*_, the pseudoeutectic is found at approximately 20–25 mol % azelate, indicating strong asymmetry; in contrast, in OG_*x*_OAz_*y*_, it shifts to around 35–45 mol % azelate (Figure 10b), much closer to equal molar ratios and thus more symmetric.

The origin of this difference lies in repeat-unit periodicity: increasing diol length reduces ester-group density and increases conformational flexibility, thereby making the two lattices more similar in their packing requirements.

Structural and thermodynamic analyses support this interpretation. Systems with shorter diols show greater variations in *d*-spacing with composition and higher defect-free energies, indicating more constrained lattice accommodation. In contrast, longer diols decrease lattice distortion during comonomer insertion and reduce the energetic penalty for defect incorporation, thereby enhancing mutual compatibility. In this sense, effective repeat-unit periodicity provides a more useful descriptor than local segment mismatch alone.

#### 6.1.2 Random Copolymers of Aromatic Polyesters

##### 6.1.2.1 Aromatic Copolyesters: Rigid Units and Isodimorphic Crystallization

Aromatic polyesters are created by combining aliphatic diols and aromatic diacids, thus maintaining the same diol/diacid structure discussed for aliphatic systems. However, unlike fully aliphatic copolyesters, where structural variation mainly involves changing the length of methylene sequences, aromatic systems allow the chemical nature of the repeating unit to be modified while keeping the backbone architecture unchanged. This type of variation is particularly easy to achieve in polyesters and is seldom possible in other polymer families, where synthetic limitations restrict systematic changes to rigid units within similar frameworks.

In these copolymers, both comonomers contain aromatic rings, but they are not geometrically equivalent. Terephthalate (T) has a single benzene ring, while 2,6-naphthalate (N) features two fused aromatic rings, resulting in a larger steric volume, greater rigidity, and a longer repeat-unit periodicity. As a result, even when the diol segment is the same, these units do not easily fit into a common crystalline lattice under typical conditions. Therefore, crystallization occurs through isodimorphism, with each component forming its own parent-like crystalline phase and only limited incorporation of the second comonomer.

##### 6.1.2.2 Effect of Aromatic Unit Geometry: Terephthalate vs Naphthalate

This behavior is consistently observed in systems such as poly­(propylene terephthalate-*ran*-propylene 2,6-naphthalate) (PT_*x*_PN_*y*_),^54^ poly­(butylene terephthalate-*ran*-butylene 2,6-naphthalate) (BT_*x*_BN_*y*_),^55^ and poly­(octamethylene terephthalate-*ran*-octamethylene 2,6-naphthalate) (OT_*x*_ON_*y*_)^56^ (Figure 11a), where the aromatic diacid is fixed and only the diol is varied. In all cases, crystallization occurs via two competing crystalline phases, and the *T*_m_-composition diagrams exhibit clear pseudoeutectic behavior. Importantly, the position of the pseudoeutectic point varies significantly with the diol length: in PT_*x*_PN_*y*_ (diol: 3 CH_2_), it is located at ∼50–55 mol % PN,^54^ in BT_*x*_BN_*y*_ (diol: 4 CH_2_), it shifts toward ∼35 mol % BN,^55^ and in OT_*x*_ON_*y*_ (diol: 8 CH_2_), it moves to ∼20–25 mol % ON,^56^ indicating strong asymmetry toward the terephthalate-rich side.

**Figure 11:**
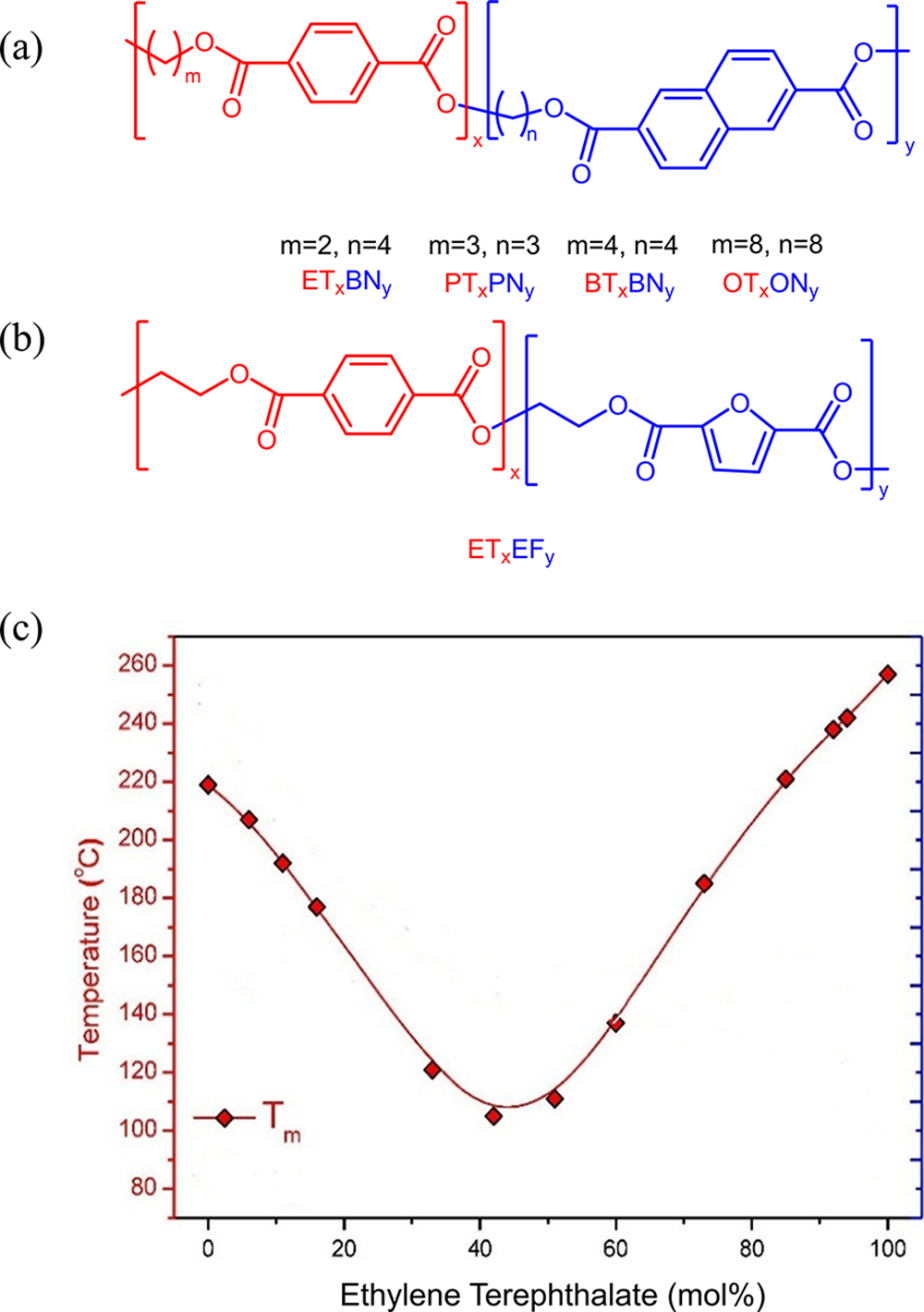
(a) Chemical structures for (a) Terephthalate-naphthalate- and (b) Terephthalate-furanoate-based copolymers. (c) Example of the symmetric pseudoeutectic point for terephthalate-based systems. Figure 11 illustrates how aromatic rigidity and spacer length modulate pseudoeutectic asymmetry without altering the system’s fundamentally isodimorphic character. Figure 11 is adapted from refs 55–57 . Copyright 2000 American Chemical Society; Copyright 2009 and 2017 Elsevier.

At first glance, the more symmetric behavior observed for shorter diols (3 CH_2_) could be interpreted as improved incorporation of the naphthalate unit into the terephthalate lattice. However, this interpretation is misleading because these systems remain strictly isodimorphic. What changes is not the crystallization mode itself, but rather the extent to which the intrinsic mismatch between the two aromatic lattices is expressed.

Shorter diols increase the density of ester groups and restrict chain packing, thus reducing the impact of the geometric mismatch between terephthalate (single aromatic ring) and 2,6-naphthalate (fused aromatic rings). As a result, both parent-like lattices become more similar in stability, leading to a more balanced competition and a pseudoeutectic closer to equal molar compositions. Conversely, longer diols (e.g., 8 CH_2_) allow greater conformational freedom and spacing between aromatic units, enabling each lattice to better express its intrinsic geometry. This enhances the mismatch between the two crystalline phases, resulting in a strongly asymmetric pseudoeutectic.

This contrasts with many fully aliphatic systems, where longer methylene sequences often promote more symmetric isodimorphism by softening local mismatch. In aromatic systems, longer flexible spacers do not erase mismatch; instead, they reveal it more clearly. This distinction highlights that pseudoeutectic position cannot be interpreted independently of the structural origin of the mismatch.

##### 6.1.2.3 Extension to Heteroaromatic Systems: Terephthalate vs Furanoate

Additional structural complexity comes from the polymorphism of the naphthalate-based component, especially in systems like OT_*x*_ON_*y*_, where different crystalline forms can appear depending on the composition and thermal history. Moreover, the inclusion of comonomer is naturally asymmetric, with the bulkier naphthalate unit being less easily incorporated into the terephthalate lattice than the reverse. This reinforces the displacement of the pseudoeutectic toward terephthalate-rich compositions in systems with longer diols.

A similar crystallization framework appears in heteroaromatic systems such as poly­(ethylene terephthalate-*ran*-ethylene furanoate) (ET_*x*_EFu_*y*_),^57^ where the diol remains ethylene (2 CH_2_) and the aromatic unit shifts from terephthalate to furanoate. Despite differences in ring structure, symmetry, and electronic properties, this system also shows isodimorphic behavior, with a pseudoeutectic minimum around 35–40 mol % EF and broad control of *T*_m_s. This behavior aligns with the structural differences between the aromatic units, which do not support the formation of a common crystalline lattice under these conditions.

Aromatic random copolymers represent a limiting case in which crystallization is governed less by gradual conformational matching and more by the inability of rigid, nonequivalent units to share a common packing motif. These systems show that lattice rigidity can preserve isodimorphism even when composition strongly modulates asymmetry.

##### 6.1.2.4 Role of the Diol Segment in Aromatic Copolyesters

A complementary perspective arises when the aromatic unit remains constant and only the diol segment varies. This is demonstrated by poly­(ethylene 2,6-naphthalate-*ran*-butylene 2,6-naphthalate) copolymers, EN_*x*_BN_*y*_, where both repeat units contain the same aromatic diacid (2,6-naphthalate), but the diol ranges from ethylene (2 CH_2_) to butylene (4 CH_2_).^58^

Despite this change, the system still shows clear isodimorphic behavior throughout the entire composition range, with a transition between PEN-type and PBN-type crystals. However, thermodynamic analysis uncovers a marked asymmetry in defect incorporation. The inclusion of bulkier butylene units into PEN crystals is much more energetically unfavorable than the reverse, indicating a distinct directional preference in comonomer incorporation.

This result shows that even in aromatic systems, where the rigid unit largely defines crystal packing, the diol segment still controls defect tolerance and directional inclusion. Accordingly, asymmetry in aromatic copolyesters is not dictated solely by aromatic mismatch, but by the combined effects of ring geometry, spacer flexibility, and repeat-unit architecture.

#### 6.1.3 Lactone-Based Copolyesters: Ring Size, Ester Density, and the Limits of Conformational Compatibility

Polylactones form a specific subgroup of aliphatic polyesters where the repeat unit is fully defined by the cyclic monomer. Unlike traditional copolyesters, where diols and diacids can be varied independently, the structure of polylactones is inherently limited by ring size, which directly sets the *n*_CH2_ in the backbone.

Importantly, not all lactones are equally suitable for copolymer synthesis. Small-ring lactones, especially γ-lactones^59^ (γ-butyrolactone (BL) with three methylene units (3 CH_2_)), show low or even unfavorable thermodynamic driving force for ring-opening polymerization because of their reduced ring strain. As a result, incorporating these into well-defined high-molecular-weight random copolymers is difficult and often leads to deviations from ideal randomness. Therefore, most experimentally relevant polylactone copolymers are made from monomers with *n*_CH_2__ ≥ 4, such as ε-caprolactone (CL), which has five methylene units (5 CH_2_);^20,47,48,60,61^ δ-valerolactone (VL), with four (4 CH_2_);^60^ ω-dodecalactone (DDL), with 12 (12 CH_2_); and ω-pentadecalactone (PDL), containing 14 (14 CH_2_),^20,47,48,61–63^ as shown in Figure 12a. Within this accessible compositional space, increasing the methylene sequence length enhances the prevalence of polymethylene segments along the chain, encouraging packing patterns that start to resemble PE-like structures. In this regime, differences in methylene sequence length (Δ*n*_CH_2__) become a key factor influencing crystallization behavior, though their predictive power may be limited when additional chemical modifications are introduced.

**Figure 12:**
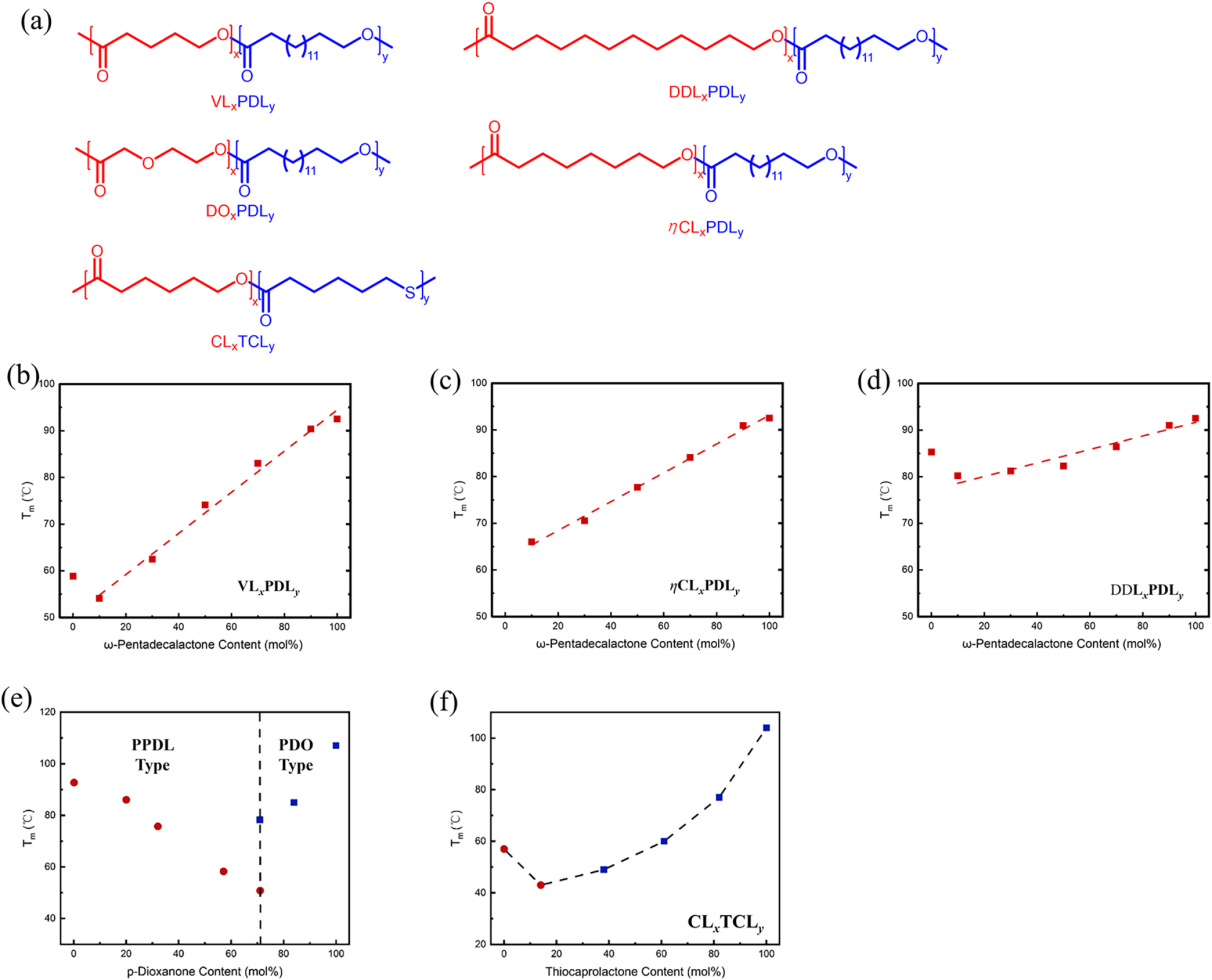
(a) Chemical structures of lactone-based copolymers; (b–d) variations of *T*_m_ vs PPDL content with *n*_CH_2__ of the other lactone showing a strongly asymmetric behavior, and in (e) with DO comonomer showing a more symmetric pseudoeutectic point. In (f) *T*_m_ vs TCL content is plotted, reflecting how the isodimorphic behavior is affected by the introduction of a sulfur atom. Figure 12 demonstrates that chemical modifications beyond methylene mismatch, including heteroatom incorporation and variations in ester density, strongly affect pseudoeutectic asymmetry and crystallization behavior. Figure 12 is adapted from refs 62–64 . Copyright 2015 and 2007 American Chemical Society; Copyright 2011 WILEY-VCH.

Large-ring lactones feature long polymethylene chains and form PE-like crystal packing. Conversely, smaller lactones increase ester group density, disrupting chain conformation and packing efficiency. While Δ*n*_*CH2*_ effectively captures conformational mismatch in purely aliphatic systems, its predictive ability diminishes when chemical modifications change the backbone functionality.

##### 6.1.3.1 Conformational Compatibility and Sensitivity to Δ*n*_CH_2__

Within polylactones made up of chemically similar repeat units, Δ*n*_CH_2__ serves as a useful descriptor to explain crystallization behavior. However, even small differences in methylene sequence length can greatly influence how the lattice accommodates them.

A representative example is CL_*x*_VL_*y*_, where Δ*n*_CH_2__ = 1 (CL: 5 CH_2_; VL: 4 CH_2_).^60^ Despite the close structural similarity between the two units, a pseudoeutectic minimum appears at about 50 mol % VL, with a minimum *T*_m_ around 20 °C, which is significantly lower than that of the corresponding homopolymers. This behavior illustrates classical isodimorphism and underscores how crystallization is affected by subtle conformational mismatches, even within purely aliphatic polyester backbones.

Early studies on copolymers based on PDL combined with other lactones reported high crystallinity across the entire composition range and a roughly linear decrease in *T*_m_ as the composition changes (Figure 12b–d). These systems are usually produced as truly random copolymers because of extensive transesterification during polymerization, which allows a direct link between composition and crystallization behavior.

For instance, in PDL_*x*_CL_*y*_ (Δ*n*_CH_2__ = 9), WAXS results show a continuous change in the crystal lattice (Figure 7) between the two homopolymers.^47,48^ However, this change is marked by the gradual loss of reflections related to chain periodicity, indicating increasing disruption of ester group ordering along the chain axis. Despite this structural disorder, relatively high crystallinity persists across a wide composition range, supporting the presence of a dominant crystalline phase.

Although such behavior has historically been interpreted as isomorphism,^47,48^ it is more accurately described as high conformational compatibility, in which a single crystalline framework accommodates compositional changes as disorder increases.

More recent work, as discussed in Section 5, using broader compositional coverage revealed a highly asymmetric pseudoeutectic minimum at very high CL content and showed that PCL-like crystals only appear at the most CL-rich compositions, whereas PPDL-type crystals dominate most of the range.^20^ This reassessment is particularly important because it demonstrates how a system can appear isomorphic when composition is undersampled, yet prove to be strongly asymmetric isodimorphic once the full range is examined.

These results show that apparent linear thermal property trends do not necessarily indicate true isomorphic crystallization, and that structural characterization is needed to determine the underlying crystallization mode, as discussed above (Section 5).

##### 6.1.3.2 Intrinsic Chemical Perturbations within ROP-Derived Systems

Deviations from compatibility cannot be fully explained by Δ*n*_CH_2__ alone. Changes in the chemical structure of the repeat unit may cause intrinsic disruptions that fundamentally change chain conformation and crystal packing.

A good example is poly­(PDL-*ran*-*p*-dioxane), PDL_*x*_DO_*y*_ (Figure 12a), where PDL has long polymethylene chains (*n*_CH_2__ = 14), while *p*-dioxanone (DO) adds a much shorter segment with only three methylene groups separated by an ether linkage. Although both monomers are polymerized via ROP polymerization, their repeat units differ significantly.^63^ While lactones create continuous polymethylene sequences interrupted by ester groups, DO incorporates an ether functionality into the backbone, effectively forming a polyester-ether structure.

This chemical modification results in distinct crystal structures and classic isodimorphic behavior, with a pseudoeutectic minimum at about 70 mol % DO,^63^ (Figure 12e) demonstrating how backbone chemistry can surpass conformational compatibility.

A more subtle change is observed in poly­(thiocaprolactone-*ran*-caprolactone) TCL_*x*_CL_*y*_ (Figure 12a) random copolymers,^64^ where thionation replaces the ester group of *ε*-caprolactone with a thioester while mostly maintaining the overall backbone structure. In this system, the *T*_m_ rises from about 43 °C at 14 mol % (the pseudoeutectic point) TCL to 77 °C at 82 mol % TCL, compared to 57 °C for PCL and 104 °C for PTCL. Notably, copolymers with more than 60 mol % TCL already show *T*_m_s higher than that of PCL, indicating improved crystallization ability.^64^

However, without detailed structural characterization, the exact crystallization mode cannot be clearly determined. Nevertheless, assuming mainly isodimorphic behavior, the location of the pseudoeutectic point offers useful insight into comonomer inclusion. In this system, even though both comonomer units have the same methylene sequence length, the pseudoeutectic composition is heavily shifted toward the *ε*-caprolactone-rich side (∼14 mol % CL in Figure 12f). This strong asymmetry suggests that *ε*-caprolactone units are more easily incorporated into the crystalline lattice of the modified component than the other way around. Because methylene-sequence length is unchanged, this asymmetry must arise from altered intermolecular interactions and modified lattice tolerance induced by heteroatom substitution. Lactone-based systems show that conformational compatibility is only part of the story. Even in frameworks that are chemically close and synthetically uniform, ester density and local backbone chemistry can decisively shift the balance between apparent compatibility, strong asymmetry, and clear isodimorphism.

#### 6.1.4 Hybrid Diol/Diacid-Lactone Copolyesters: Structural Competition and Continuous Transitions between Crystallization Modes

Random copolyesters that combine lactone-derived units with traditional diol/diacid–based repeat units serve as a particularly insightful example, because they naturally introduce two fundamentally different chain architectures within a single polymer backbone. Unlike the systems described earlier, where variations come from changes in methylene sequence length within a consistent structural framework, here the difference also involves the distribution of ester groups and the effective periodicity of the repeat unit.

Lactone-derived units generally consist of long polymethylene chains interrupted by a single ester group, resulting in a relatively low ester-group density and conformations similar to PE packing. In contrast, diol/diacid-derived units have a higher ester density, with two ester groups per repeat unit, leading to a more restricted chain conformation and a different packing periodicity. As a result, even when the total number of methylene groups is similar, the effective repeat unit structure varies significantly, preventing the formation of a shared crystalline lattice.

Consistently, copolymers combining these two families do not show highly symmetric crystallization behavior and instead exhibit isodimorphic or mixed crystallization modes, often with noticeable asymmetry depending on composition.

A clear example is the family of poly­(alkylene succinate-*ran*-*ε*-caprolactone) copolymers, where the diol length in the succinate unit is systematically varied (ethylene (ES), butylene (BS), octylene (OS), and dodecylene (DS) succinate; *n*_CH_2__ = 2, 4, 8, and 12, respectively), while the lactone unit (CL, *n*_CH_2__ = 5) remains unchanged, as show the chemical structures in Figure 13a.^9^ Despite the increasing similarity in methylene sequence length as the diol gets longer, all systems mainly show isodimorphic crystallization behavior, confirming that matching Δ*n*_CH_2__ alone is not enough to achieve symmetric crystallization when the repeat unit structure differs.

**Figure 13:**
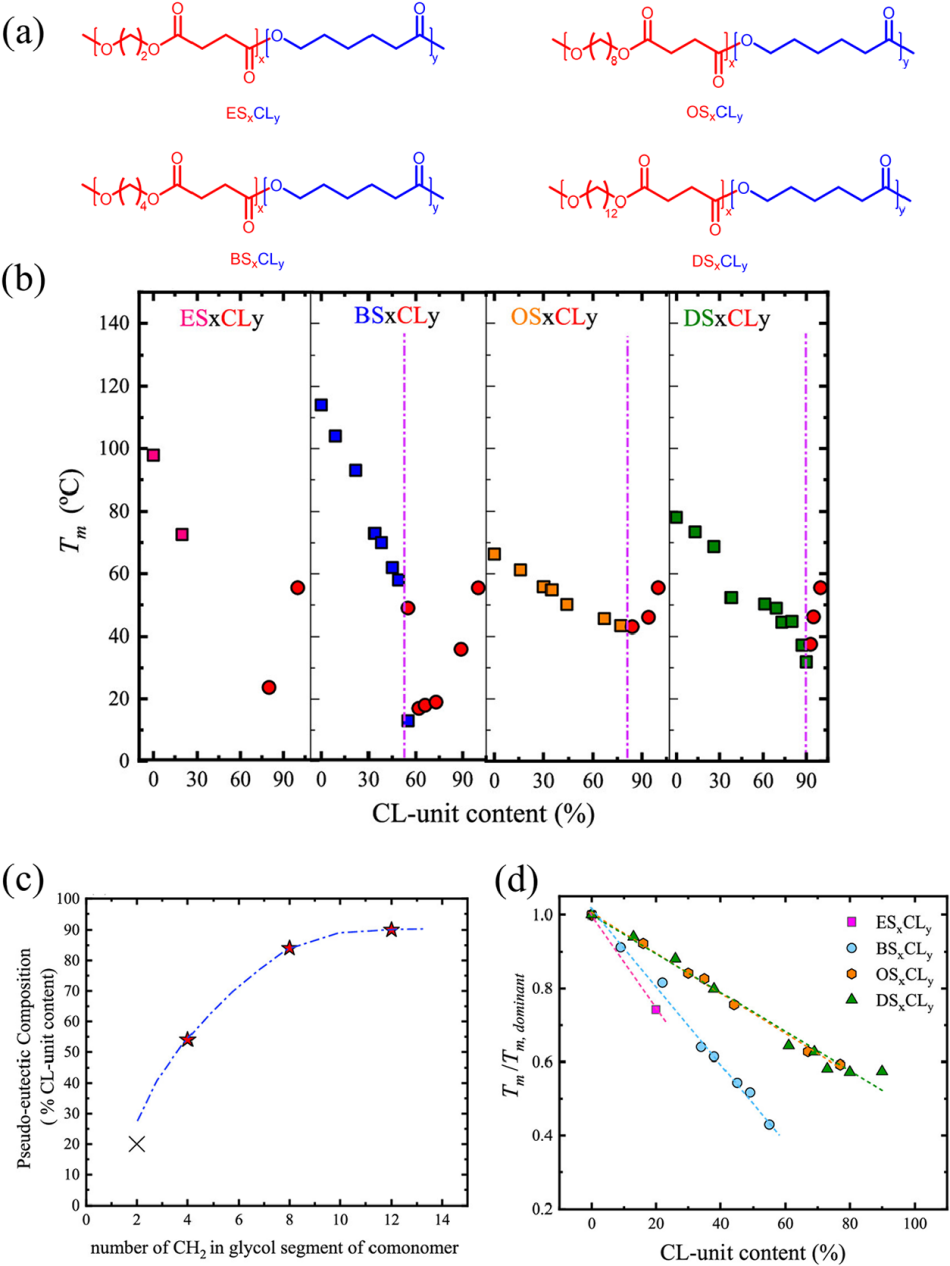
(a) Chemical structure of polysuccinates-*ran*-PCL copolymers; (b) Comparison of *T*_m_ vs comonomer content for each system; (c) Pseudoeutectic point evolution with *n*_CH_2__ in the succinate segment, and (d) Normalized *T*_m_ as a function of the CL-content. Figure 13 illustrates how progressively reducing architectural mismatch shifts the balance from exclusion-dominated to more symmetric isodimorphic behavior without achieving true crystallographic equivalence. Figure 13 is adapted from ref 9. Copyright 2024 American Chemical Society.

Importantly, this series demonstrates a systematic evolution of the pseudoeutectic composition based on chain architecture. The shortest diol system, ES_*x*_CL_*y*_ (with *n*_CH_2__ = 2 versus 5), shows a mixed isodimorphism/total exclusion behavior, with an amorphous region around 52–56 mol % CL, indicating poor mutual accommodation between the two crystalline phases. Conversely, systems with longer diols, such as BS_*x*_CL_*y*_, OS_*x*_CL_*y*_, and DS_*x*_CL_*y*_, display isodimorphic behavior across the entire composition range, with a pseudoeutectic point that shifts (Figure 13b,c) from highly asymmetric (exclusion/isodimorphism case) to more balanced compositions (∼50% CL in BS_*x*_CL_*y*_) and then shifts again (to about 80% CL in OS_*x*_CL_*y*_ and DS_*x*_CL_*y*_), reflecting a gradual change in the relative crystallization ability of each component.^9^

The main mechanistic point is that increasing diol length gradually reduces the architectural mismatch by lowering ester-group density in the diol/diacid segment, but does not eliminate the fundamental difference between the two repeat-unit types. Thus, compatibility improves, judging by a lower decrease in *T*_m_ (Figure 13d), yet true equivalence between the competing phases is not achieved.

This behavior closely resembles that seen in pure diol/diacid systems like poly­(hexamethylene succinate-*ran*-longer diacids), where changing one segment (diacid or diol) shifts the pseudoeutectic point by modifying the overall chain periodicity rather than a single local mismatch. In both cases, the pseudoeutectic composition indicates a balance among chain flexibility, ester-group density, and packing needs.

### 6.2 Random Copolycarbonates: Structural Regimes, Pseudoeutectic Behavior, and Crystallization Complexity

Random aliphatic copolycarbonates based on α,ω-diols with varying methylene sequence lengths serve as a valuable model system for studying crystallization in semicrystalline polymers. These materials are generally synthesized through melt polycondensation of diols with carbonate sources, resulting in linear chains where the number of methylene units can be systematically adjusted while keeping a constant carbonate backbone. Consequently, copolycarbonates offer a well-defined structural platform that allows for more direct investigation of how chain geometry influences crystallization compared to other polymer families.

In the literature, most studies on random copolycarbonates have focused on changing the length of the methylene sequence in the diol segment while keeping the carbonate unit the same.^5–8,65,66^ This has allowed researchers to establish clear structure-property relationships, especially in systems where the parent homopolymers differ only in the number of methylene groups. At the same time, it should be noted that modifying the carbonate part presents an additional, largely unexplored degree of freedom that could further expand the crystallization design space.

A key feature of aliphatic polycarbonates is the existence of two structural crystallization regimes based on methylene sequence length: an even-odd region (usually *n*_CH_2__ = 6–9) and a saturation region (*n*_CH_2__ ≥ 10).^67,68^ In the even-odd region, crystallization is strongly affected by the carbonate groups, causing alternating thermal behavior, differences in crystal structure, and increased sensitivity to comonomer incorporation. Conversely, in the saturation region, the impact of the carbonate groups diminishes, and crystallization is increasingly influenced by methylene packing, leading to PE-like conformations and more consistent trends in thermal properties. This duality adds an extra layer of structural control that is generally not available in polyester systems.

Within this framework, random copolycarbonates show a wide range of crystallization behaviors depending on the relative chain lengths of the comonomer units. Classical isodimorphic behavior has been reported in several families, including C4_*x*_C7_*y*_,^65^ C4_*x*_C10_*y*_,^69^ C4_*x*_C12_*y*_,^65^ C10_*x*_C8_*y*_,^66^ and C7_*x*_C12_*y*_ ^65^ systems, where crystallization occurs across the full composition range with a characteristic pseudoeutectic minimum in the *T*_m_. Despite exhibiting a pseudoeutectic trend, mixed crystallization modes have been reported in C4_*x*_C8_*y*_,^7^ C6_*x*_C8_*y*_,^5,6,8^ C7_*x*_C8_*y*_,^6,8^ and C12_*x*_C8_*y*_.^6,8^ Selected chemical structures of these copolymers are shown in Figure 14a.

**Figure 14:**
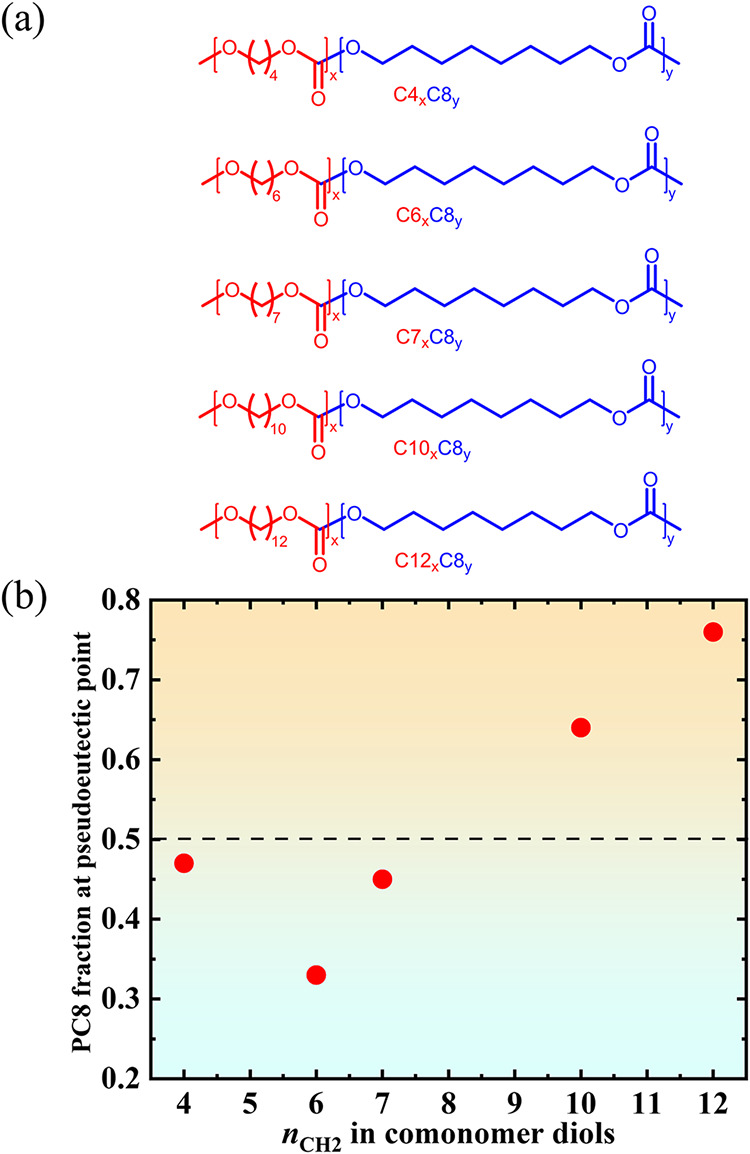
(a) Chemical structures of PC8-based copolymers. (b) Pseudoeutectic point position variation of PC8-based systems, in which the *n*_CH2_ of the other comonomer was varied (*n*_CH_2__ = 4, 6, 7, 10, and 12). Figure 14 summarizes how mixed crystallization modes and pseudoeutectic asymmetry evolve across PC8-based copolycarbonates as a function of methylene-sequence length and structural regime. Figure 14 is adapted from refs 5–7 and 67. Copyright 2023, 2024, and 2025 American Chemical Society; Copyright 2025 Elsevier.

So far, mixed crystallization modes have been observed in PC8-based copolymers, specifically isodimorphism/isomorphism for C6_*x*_C8_*y*_,^5,6,8^ C7_*x*_C8_*y*_,^6,8^ and C12_*x*_C8_*y*_,^6,8^ as well as isodimorphism/isomorphism/total exclusion for C4_*x*_C8_*y*_,^7^ as discussed in Section 3. Beyond simply identifying these mixed modes, an important parameter that emerges is the compositional window over which the γ phase remains stable, offering additional insight into the balance between comonomer inclusion and exclusion.

For isodimorphic or isomorphic systems, the γ phase generally appears over a range of compositions that can vary widely depending on the structural relationship between the comonomer units. Typically, for intermediate chain lengths (e.g., C6_*x*_C8_*y*_), relatively broad γ-phase ranges are observed, which corresponds with improved conformational compatibility and efficient comonomer incorporation. However, even small structural changes can significantly affect this behavior. For instance, in C7_*x*_C8_*y*_ copolymers, the introduction of odd-even asymmetry results in a noticeable narrowing of the γ-phase range, showing reduced packing efficiency and decreased stability of the cocrystalline structure.

As the length of the methylene sequence increases further (e.g., C12_*x*_C8_*y*_), the compositional window of the γ phase becomes more limited and, in some cases, difficult to measure experimentally. This narrowing reflects the growing dominance of the longer comonomer, which has a higher crystallization ability that favors self-crystallization over cocrystallization. In this context, the absence of clearly reported mixed modes in C10_*x*_C8_*y*_ ^66^ should not necessarily be seen as purely isodimorphic behavior, but rather as a consequence of a very narrow compositional window that may go unnoticed if only limited compositions are tested.

Importantly, this trend is not strictly monotonic throughout the complete compositional space. The C4_*x*_C8_*y*_ system is a unique case, where a relatively broad γ-phase window appears under nonisothermal conditions despite the significant structural mismatch between comonomers. However, this perceived stability heavily relies on crystallization conditions. Under isothermal crystallization, the γ-phase window in C4_*x*_C8_*y*_ copolymers narrows substantially (Figure 3g), indicating that its stability is at least partially kinetically controlled. This behavior suggests that similar γ-phase windows can arise from fundamentally different mechanisms: enhanced structural compatibility in systems like C6_*x*_C8_*y*_, or kinetic stabilization under conditions that restrict comonomer exclusion in systems such as C4_*x*_C8_*y*_.

Indeed, combining comonomers from different structural regimes adds an extra layer of complexity. Copolymers made from components within the even-even region (e.g., C6_*x*_C8_*y*_) often show strong structural competition and a higher tendency for cocrystallization, which helps form new crystalline phases across a broader range of compositions. In contrast, combinations involving odd-even pairs (e.g., C7_*x*_C8_*y*_) introduce a conformational mismatch that limits both the extent and stability of cocrystallization. When one component is in the saturation regime (e.g., C12_*x*_C8_*y*_), crystallization becomes more asymmetric, and the compositional range for cocrystallization shrinks or becomes difficult to observe experimentally.

Overall, these observations show that crystallization in copolycarbonates is influenced not only by Δ*n*_*CH2*_ but also by the interplay among geometric fit, conformational effects, crystallization ability, and kinetic constraints. All these factors determine both the presence and the stability range of additional crystalline phases.

Besides the occurrence of mixed crystallization modes, the copolycarbonate family also provides important insights into the development of the pseudoeutectic point. When using PC8 as a reference comonomer, a general shift of the pseudoeutectic composition toward higher PC8 contents is observed as the length of the second comonomer increases, as shown in Figure 14b. However, this trend is not monotonic, and more complex behavior occurs at shorter chain lengths.

For instance, in C4_*x*_C8_*y*_ copolymers, the pseudoeutectic composition occurs at about 47 mol % PC8, while in C6_*x*_C8_*y*_ it decreases significantly to around 33 mol % PC8, making it one of the most asymmetric cases in the family. As the chain length increases further (C7, C10, C12), the pseudoeutectic point gradually shifts toward higher PC8 contents, reaching approximately 45 mol % PC8 for C7_*x*_C8_*y*_ and exceeding 60–75 mol % PC8 for longer comonomers such as C10 and C12.

Instead of reflecting experimental uncertainty related to mixed crystallization modes, this nonmonotonic pattern indicates a transition between competing structural factors. In the case of C6_*x*_C8_*y*_, the pronounced asymmetry suggests an optimal balance between conformational compatibility and intermolecular interactions, which enables efficient incorporation of the shorter comonomer into the PC8 crystalline lattice. Conversely, the more symmetric behavior observed in C4_*x*_C8_*y*_ cannot be attributed to improved compatibility but instead signifies a different regime where significant structural mismatch decreases cocrystallization efficiency. As the chain length surpasses C8, the crystallization ability of the longer comonomer becomes more dominant, gradually shifting the pseudoeutectic composition toward mixtures richer in PC8.

This behavior shows that the position of the pseudoeutectic point is affected by a balance between intermolecular interactions and chain length effects. At moderate chain lengths, intermolecular interactions and conformational matching mainly control the behavior, leading to very asymmetric pseudoeutectic compositions. On the other hand, at longer chain lengths, the increased crystallization ability linked to extended polymethylene sequences outweighs these effects, causing the system to favor crystallization mainly influenced by the longer component.

Copolycarbonates provide the clearest current demonstration that random copolymer crystallization is best understood as a continuum of structurally tunable states. More than any other family discussed here, they show how small changes in chain architecture can reorganize the balance among parent-like crystallization, local cocrystallization, exclusion, and kinetic trapping.

### 6.3 Random Copolyamides: Coupling between Isodimorphism, Polymorphism, and Hydrogen-Bond-Driven Constraints

Compared with polyester- and polycarbonate-based systems, random copolyamides display more complex crystallization behavior because strong, directional hydrogen bonding couples crystal packing to a cooperative intermolecular network. As a result, comonomer incorporation does not simply distort a lattice; it also perturbs hydrogen-bond periodicity, polymorphic stability, and solid-solid transitions.

Polyamides may be broadly grouped into AB-type systems, such as PA6, which contain one amide group per repeat unit, and AABB-type systems, such as PA66, PA56, or PA88, which contain two. This distinction is not merely nominal: it changes the density and spatial distribution of hydrogen bonds along the chain and therefore the extent to which a crystal can tolerate comonomer incorporation without losing its cooperative network. For this reason, crystallization in random copolyamides is governed simultaneously by packing constraints and by the need to preserve hydrogen-bond regularity.

In representative systems like A66_*x*_A88_*y*_ ^70^ and A6_*x*_A56_*y*_ ^71^ random copolymers (Figure 15a), a typical isodimorphic behavior is observed, characterized by pseudoeutectic trends in melting and crystallization temperatures and the formation of composition-dependent crystalline phases.^70,71^ In these systems, partial inclusion of comonomer units into the host crystal lattice occurs, with a clear asymmetry depending on the relative stability of each crystalline phase. For example, in A6_*x*_A56_*y*_ copolymers, the incorporation of A56 units into the PA6 lattice is less energetically favorable than the reverse, leading to preferential crystallization of PA56-rich phases and a pseudoeutectic minimum at around 30 mol % of PA56.^71^

**Figure 15:**
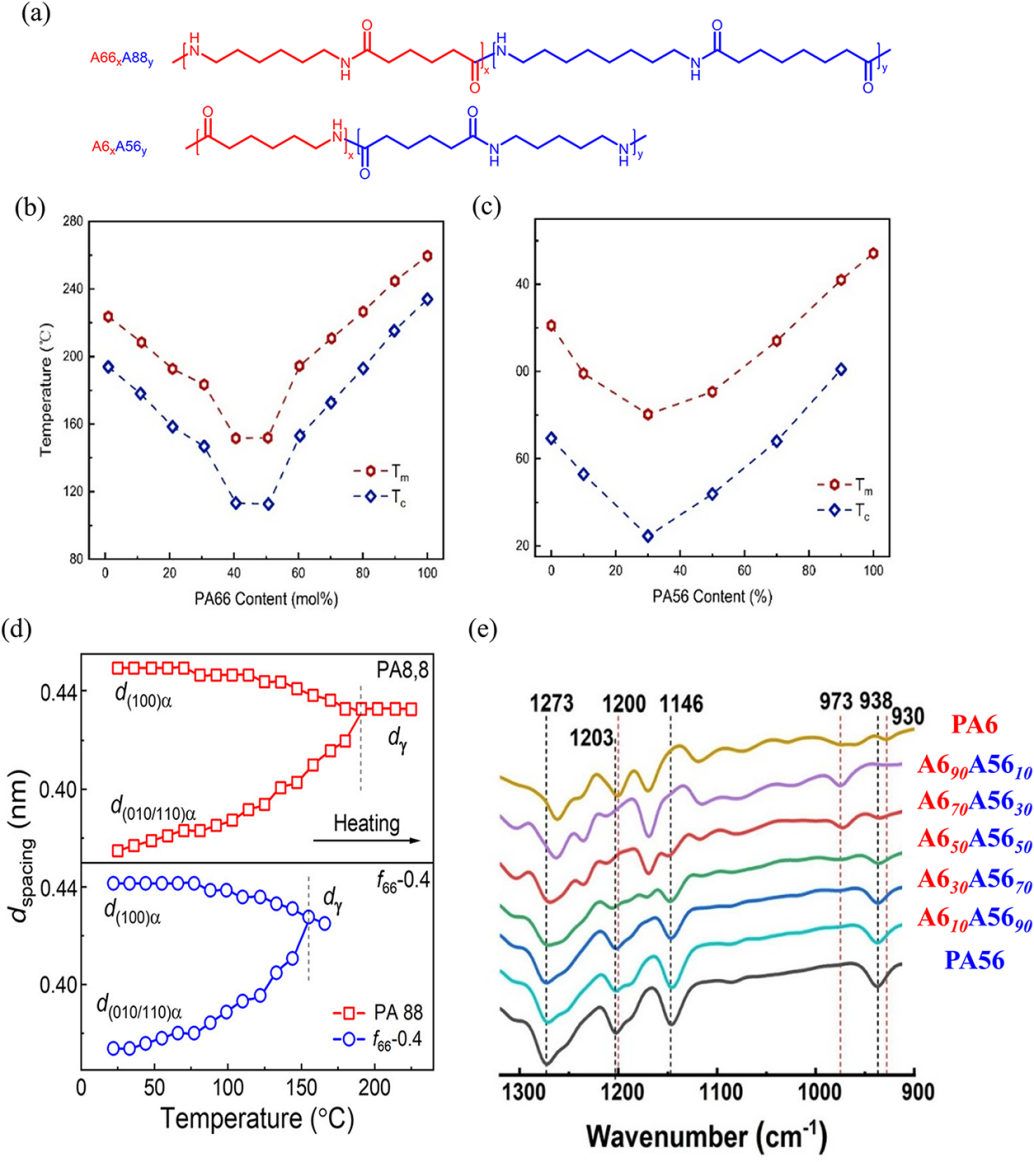
(a) Chemical structures of A66_*x*_A88_*y*_ and A6_*x*_A56_*y*_ copolyamides. Temperature vs comonomer content variation for (b) A66_*x*_A88_*y*_ and (c) A6_*x*_A56_*y*_ copolyamides. For the latter, (e) conformational differences are also detected by FTIR experiments. The Brill-like transition is detected by the (d) variation of *d*-spacing vs temperature. Figure 15 illustrates how hydrogen-bond periodicity and polymorphic accessibility introduce additional constraints that strongly influence pseudoeutectic asymmetry and solid-solid transitions in random copolyamides. Figure 15 is adapted from refs 70 and 71. Copyright 2022 American Chemical Society; Copyright 2023 Elsevier.

This contrast becomes especially clear when A66_*x*_A88_*y*_ and A6_*x*_A56_*y*_ are compared directly (Figure 15). In the former, the two comonomers share a more similar hydrogen-bond density and repeat-unit periodicity, so mutual accommodation can be achieved with moderate lattice distortion, and symmetric pseudoeutectic behavior (Figure 15b). In the latter, PA6 and PA56 differ not only in methylene sequence length but also in amide-group density and topology, producing a much larger energetic penalty for reciprocal inclusion, reflected in a more asymmetric pseudoeutectic behavior (Figure 15c).

Unlike in polyesters or polycarbonates, the effect of comonomer incorporation in polyamides frequently extends into polymorphism. Aliphatic polyamides can crystallize in α and γ forms (Figure 15d,15e), and the balance between them is linked by the Brill transition.^72^ In random copolyamides, incorporation of a second unit perturbs both conformation and hydrogen-bond ordering, so that the γ → α transition may become irreversible or even be suppressed near the pseudoeutectic region.^70,73^ At higher crystallization temperatures, where comonomer inclusion is reduced, more regular hydrogen-bonded structures can develop and reversibility may be restored. This means that, in polyamides, comonomer incorporation affects not only which lattice forms, but also whether subsequent solid-solid reorganization remains accessible.

Additional complexity appears when even-even and odd-even polyamide units are combined. In these systems, odd-even sequences tend to stabilize the γ phase and hinder reversion to α, whereas even-even systems more readily support reversible γ ↔ α transitions.^74^ When both are incorporated into the same random copolymer, the outcome depends strongly on composition and crystallization conditions, because hydrogen-bond periodicity and conformational matching are disrupted simultaneously. At the molecular level, the central constraint is that packing and hydrogen bonding cannot be optimized independently. Once comonomer incorporation alters the spacing or orientation of amide groups, the system must trade hydrogen-bond regularity against conformational accommodation. This competition helps explain why strict isomorphism is not observed in random copolyamides: maintaining a truly common lattice would require preserving hydrogen-bond periodicity despite continuous structural substitution, which is highly unlikely.

Finally, the relatively limited number of reported copolyamide systems is unlikely to reflect only synthetic difficulty. It also suggests that the structural window allowing partial comonomer incorporation without catastrophic disruption of hydrogen-bond regularity is intrinsically narrow. For this reason, polyamides provide a particularly stringent test of the continuum concept: they remain largely within the isodimorphic regime, but reveal that crystallization modes may also be coupled to polymorphic and solid-solid transitions rather than to inclusion/exclusion behavior alone.

## 7 Applications, Concluding Remarks, and Perspectives

The discussion above suggests that crystallization modes in random copolymers should not be treated merely as descriptive categories. Rather, they should be viewed as tunable structural states that can be accessed, shifted, or combined through molecular design and processing history. From this perspective, crystallization is not simply a consequence of chemical structure but an active variable that governs how molecular heterogeneity is translated into macroscopic performance.

### 7.1 Applications: Regulating Properties through Crystallization Modes

Although systematic property studies remain relatively rare, especially beyond isodimorphic systems, existing results already show that crystallization modes offer a powerful way to control key material properties.^53,60,75–79^ In isodimorphic copolymers, the coexistence of crystallization across the entire composition range and partial comonomer inclusion allows for a continuous tuning of *T*_m_, crystallinity, and lamellar organization. This pseudoeutectic behavior is especially beneficial for processing, as it enables lowering the softening temperature or *T*_m_ while maintaining semicrystallinity.

This balance is particularly important in applications like hot-melt adhesives,^80^ where a low processing temperature must be paired with enough cohesive strength when solid. In this context, isodimorphic copolymers offer a way to adjust adhesion and cohesion through crystallization behavior rather than relying solely on changes in chemical composition.

At the mechanical level, the relevance of crystallization modes extends beyond simple changes in crystallinity. In several isodimorphic copolyesters, Young’s modulus and strength exhibit pseudoeutectic-like minima, whereas elongation at break shows the opposite trend and can surpass that of the parent homopolymers near the pseudoeutectic region.^53,60,75–79^ These trends indicate that mechanical behavior is governed not only by the crystalline fraction but also by how comonomer incorporation reorganizes defect density, lamellar thickness, and the connectivity between crystalline and amorphous regions.

Another important consequence of isodimorphic crystallization is the increase in nucleation density near the pseudoeutectic composition. This typically leads to the formation of smaller spherulites, thereby generating a finer semicrystalline microstructure. When the characteristic size of these structures approaches or falls below the wavelength of visible light, light scattering is reduced, and optical transparency improves. Accordingly, pseudoeutectic compositions may offer a practical route to improve transparency without fully sacrificing crystalline order.

Barrier and transport properties are also expected to be strongly influenced by crystallization mode. Variations in lamellar thickness, crystal perfection, and interlamellar amorphous structure modify diffusion pathways and therefore affect permeability. More defective and less regular crystalline organizations may facilitate transport, whereas exclusion-dominated or highly ordered parent-like crystals are expected to act as more effective barriers. In this sense, crystallization mode should be regarded not merely as a structural descriptor but as a design variable for transport control. Recent work has further shown that these effects can extend to biomedical nanocarrier performance, where subtle differences in crystalline structure and crystallinity in isodimorphic biodegradable copolyesters directly influenced nanoparticle stability, drug encapsulation, and sustained-release behavior.^81^ This observation suggests that crystallization modes may provide a powerful strategy to regulate drug-matrix interactions and transport phenomena beyond conventional packaging or membrane applications.

A similar argument applies to degradability in biodegradable semicrystalline polymers. Hydrolytic and biodegradation processes depend strongly on crystallinity and on the accessibility of amorphous regions, both of which can be tuned by composition in isodimorphic systems. However, the degradation behavior is not solely governed by the crystalline fraction. The extent of comonomer inclusion and the resulting crystal structure also influence the ease with which water, enzymes, or reactive species penetrate the material. Consequently, crystallization modes are expected to affect degradation by altering crystal perfection, defect density, and amorphous-phase accessibility.

Importantly, the influence of crystallization modes is not restricted to single-phase copolymers.^82,83^ Recent studies have shown that isodimorphic copolymers can induce unusual morphologies in blends, including mixed or interpenetrating spherulitic structures.^83^ These results suggest that crystallization-driven interactions may promote mesoscale compatibility even in thermodynamically immiscible systems, opening the possibility of compatibilization through crystallization rather than solely through chemistry.

At the same time, studies of properties remain strongly skewed toward isodimorphic systems. The functional implications of local isomorphism and mixed-mode crystallization remain largely unexplored. This is a major gap, because these regimes may reorganize crystallinity in ways that cannot be captured by simple extrapolation from classical pseudoeutectic systems.

### 7.2 Perspective: Toward Crystallization-by-Design

The results discussed throughout this work support a broader framework in which crystallization modes are understood as tunable structural states shaped by the interplay of molecular architecture, intermolecular interactions, and processing conditions. In this framework, crystallization is not a fixed end point but a structurally selective process strongly influenced by thermal history and kinetics. This process ultimately determines how molecular heterogeneity is translated into morphology and macroscopic performance.

From a molecular perspective, comonomer choice influences sequence compatibility, conformational matching, and interaction strength. Together, these factors determine whether repeat units are incorporated, partially accommodated, or excluded from the crystal lattice. Differences between polymer families are particularly important in this context. Hydrogen bonding in polyamides imposes much stronger topological constraints than the weaker dipole-dipole or van der Waals interactions operating in many polyesters and polycarbonates. In addition, polymorphism, solid-solid transitions, and subtle packing constraints may strongly modify the stability of specific crystallization modes.

Mixed crystallization modes provide a particularly valuable view into this structural landscape. Rather than representing unusual exceptions, they demonstrate that crystallization behavior may evolve gradually within a single copolymer system as a function of composition or crystallization pathway. These observations make explicit that crystallization mode is not simply a thermodynamic label, but a pathway-dependent structural state that may be selected or suppressed by thermal history and kinetics. Collectively, these results support the view that crystallization in random copolymers is better understood as a continuum of interconnected structural regimes rather than as a set of strictly discrete categories. Importantly, this framework should not be interpreted as purely qualitative. Several experimentally accessible descriptors are emerging that may help position random copolymer systems within the inclusion-exclusion landscape. These include the position and symmetry of the pseudoeutectic point, the extent of lattice evolution with composition, the stability range of mixed crystalline phases, the evolution of SN *Domain II* and SSA fractionation behavior, and quantitative analyses of comonomer inclusion/exclusion. These descriptors may provide the basis for experimentally grounded, ultimately predictive structure-crystallization-property relationships.

A central challenge in advancing this framework is the quantification of comonomer inclusion. Traditional techniques such as DSC and WAXS provide robust qualitative evidence: melting-point depression, pseudoeutectic trends, and lattice distortion all indicate that comonomer incorporation affects the crystalline phase. However, these observables remain indirect and do not by themselves provide a quantitative measure of how much of a given comonomer is truly incorporated into crystal interiors.

This limitation points to the need for multiscale characterization. Solid-state NMR can probe local chemical environments and segmental proximity, providing direct information on whether comonomer units are present within crystalline regions.^84^ Advanced structural and morphological methods such as AFM can, in turn, reveal how comonomer incorporation modifies nanoscale organization, including lamellar dimensions, local heterogeneity, and phase distribution.^85^ These methods make it possible to connect local inclusion with mesoscale morphology, something that neither DSC nor diffraction can accomplish alone.

For this reason, comonomer inclusion should not be treated as a single scalar quantity, but as a multiscale phenomenon that must be resolved through combined thermal, structural, spectroscopic, and morphological analyses. In this respect, mixed-mode copolymers offer a particularly valuable opportunity. Local isomorphic windows, where both comonomers participate in a common lattice, may serve as experimentally accessible reference states approaching complete inclusion. These states could provide practical upper benchmarks against which partial inclusion in neighboring compositions may be evaluated. They may therefore help define experimental boundaries among partial inclusion, local cocrystallization, and exclusion-dominated crystallization regimes.

Another major direction is the extension of crystallization studies toward nonequilibrium and processing-relevant conditions. Conventional DSC and WAXS/SAXS remain essential tools, but sequence-sensitive techniques such as SN and SSA are critical for resolving how sequence distribution affects crystallization. In parallel, fast scanning calorimetry provides access to the high cooling-rate regimes where kinetic trapping may control phase selection. This is particularly important because some mixed-mode states may only become accessible under nonequilibrium conditions and therefore remain invisible in conventional experiments.

## 8 Concluding Remarks

The central message of this Perspective is that crystallization in random copolymers should be understood as a continuum of structurally distinct but interconnected states, rather than as a set of isolated categories. Within this framework, classical modes such as isomorphism, isodimorphism, and comonomer exclusion should be understood as dominant or limiting crystallization regimes, linked by transitional and mixed-mode behaviors. These crystallization states represent different structural solutions to the same fundamental problem: how chemically distinct repeat units are accommodated, rejected, or reorganized during crystal formation.

This shift has an important consequence: crystallization should no longer be treated as a passive outcome of composition, but as an active design parameter. By selecting comonomers, controlling composition, and tuning processing history, it should become possible to navigate the crystallization landscape deliberately and thereby regulate thermal, mechanical, optical, barrier, and degradation properties.

The emergence of mixed crystallization modes and local isomorphic states further expands this design space. At the same time, it reveals a strong asymmetry in the field: classical isodimorphic systems have been studied in considerable detail, whereas systems described as isomorphic or exclusion-dominated have often been examined with less rigorous criteria. As a result, their true structural behavior is frequently less certain than the terminology implies.

The next challenge is therefore not merely to refine classification but to establish experimentally grounded descriptors that can position random copolymer systems along the crystallization continuum. Achieving this goal will require denser compositional mapping, stronger structural evidence, broader use of sequence-sensitive methods, and systematic correlation with functional properties.

If successful, the field may move beyond empirical assignment of crystallization modes toward a true crystallization-by-design framework in which specific structural states are intentionally targeted to achieve tailored material performance across polymer families and processing conditions.
